# Effects of dietary quercetin supplementation on growth performance and intestinal function in weaned pigs challenged by rotavirus

**DOI:** 10.1016/j.aninu.2026.06.005

**Published:** 2026-09-29

**Authors:** Kang Wang, Zhentao He, Kunhong Xie, Xiang Li, Aibing Yu, Yan Li, Yuheng Luo, Ping Zheng, Hui Yan, Xiangbing Mao, Jun He

**Affiliations:** a Institute of Animal Nutrition, Sichuan Agricultural University, Chengdu 611130, Sichuan, China; b Key Laboratory of Animal Nutrition and Efficient Feed Utilization, Chengdu 611130, Sichuan, China; c Newhope Co., Ltd., Chengdu 610011, Sichuan, China

**Keywords:** Rotavirus, Weaned pig, Quercetin, Inflammation, Intestinal barrier, Apoptosis

## Abstract

Rotavirus (RV) is a major enteric pathogen that disrupts intestinal function and reduces growth efficiency in weaned pigs. Nutritional interventions may help preserve intestinal homeostasis during infection. This study evaluated whether dietary quercetin supplementation mitigates RV-induced intestinal injury in weaned pigs and validated key findings in an intestinal epithelial cell model. A total of 32 weaned pigs aged 21 d with an initial body weight of 6.49 ± 0.04 kg were randomly assigned to four dietary treatments (*n* = 8 per group) for a 17-d trial: a non-challenged control group, an RV-challenged group, and two RV-challenged groups supplemented with 100 or 200 mg/kg quercetin. In parallel, the porcine intestinal epithelial cell line IPEC-J2 cells were assigned to control, RV, RV  +  40  μmol/L quercetin, and RV  +  80  μmol/L quercetin groups to assess cell viability, apoptosis, tight junction integrity, and nuclear factor-kappa B (NF-κB) p65 nuclear translocation. Results showed that quercetin did not affect average daily feed intake or gain before challenge (*P* > 0.05), whereas 200 mg/kg quercetin significantly improved feed efficiency after RV infection (*P* = 0.017). At the systemic level, 100 mg/kg quercetin increased total protein (*P* = 0.042), while both 100 and 200 mg/kg quercetin increased albumin, red blood cell count, hemoglobin, and hematocrit (*P* < 0.05); only 200 mg/kg quercetin reduced platelet count (*P* = 0.025). Consistent with these systemic responses, 200 mg/kg quercetin decreased tumor necrosis factor-α (TNF-α) and interleukin-6 (IL-6) concentrations and increased interleukin-10 (IL-10) concentrations in both serum and jejunum relative to the RV group (*P* < 0.05). In the small intestine, under RV challenge, quercetin significantly increased villus height and villus-to-crypt ratio in the jejunum and ileum (*P* < 0.05); only 200 mg/kg increased maltase activity in the duodenum and jejunum (*P* < 0.05). Both doses increased jejunal relative *CAT1* mRNA expression, whereas only 200 mg/kg increased relative *SNAT2* and *FATP1* mRNA expression (*P* < 0.05). Moreover, quercetin attenuated RV-induced barrier dysfunction, as evidenced by reduced serum diamine oxidase (DAO) activity and D-lactate concentration and increased jejunal relative *ZO-1* and *Occludin* mRNA expression at 200 mg/kg (*P* < 0.05), while both doses enhanced ZO-1 staining, *Claudin1* expression, and secretory immunoglobulin A (sIgA) abundance (*P* < 0.05). Quercetin further attenuated RV-induced jejunal epithelial apoptosis, accompanied by decreased jejunal relative *Caspase8* and *BAX* mRNA expression at 100 mg/kg (*P* < 0.05) and decreased relative *BIM* mRNA expression at both doses (*P* = 0.009). Consistently, in IPEC-J2 cells, 40 and 80 μmol/L quercetin significantly reduced RV-induced apoptosis (*P* < 0.05), while quercetin improved cell viability, restored ZO-1 immunofluorescence, and inhibited RV-induced NF-κB p65 nuclear translocation (*P* < 0.05). These results suggest that dietary quercetin, particularly at 200 mg/kg, alleviates RV-induced intestinal dysfunction and impaired feed efficiency by modulating inflammatory responses, suppressing epithelial apoptosis, and reinforcing intestinal barrier integrity.

## 1 Introduction

Rotavirus (RV) is a major cause of acute, severe gastroenteritis in young children and animals worldwide (Cai et al., 2025; Tian et al., 2022). In intensive swine production, weaned pigs undergo rapid intestinal remodeling alongside ongoing immune maturation (de Groot et al., 2021; Han et al., 2024), a developmental window that increases susceptibility to RV challenge. Rotavirus infection not only damages the intestinal mucosa and impairs digestion and absorption but also contributes to herd-level growth retardation, reducing feed efficiency and causing substantial economic losses to the swine industry (Amimo et al., 2021; Chen et al., 2017). Given the lack of specific and scalable therapeutic options for RV infection in commercial settings, where control largely relies on supportive care and management (Patel et al., 2025), there is a practical need to develop feasible intervention strategies that mitigate RV-induced intestinal damage and facilitate the recovery of intestinal function.

Existing evidences suggest that the adverse effects of RV infection on production performance in weaned pigs are closely associated with small intestinal mucosal injury (Lv et al., 2024). After entering the gut, RV preferentially infects mature epithelial cells at villus tips, causing villus atrophy and impairing digestive and absorptive function by reducing disaccharidase activity and nutrient-transporter expression (Lorrot and Vasseur, 2007; Yan et al., 2023). Rotavirus infection also elicits robust local and systemic inflammatory responses, in part through activation of pro-inflammatory signaling pathways, thereby inducing cellular stress (Hou et al., 2025; Zhu et al., 2017). Persistent stress can promote epithelial apoptosis and disrupt tight-junction architecture, increasing intestinal permeability and contributing to systemic metabolic disturbances. Together, inflammatory amplification and epithelial homeostasis failure constitute key pathogenic features of RV infection.

In this context, plant-derived bioactive compounds have become a major focus in intestinal health research because of their generally favorable safety profiles and broad, pleiotropic biological activities (Lu et al., 2026). Quercetin, a widely distributed dietary flavonoid, has received substantial attention for its immunomodulatory and cytoprotective properties (Han et al., 2025; Manjunath and Thimmulappa, 2022). Prior studies indicate that quercetin can dampen pro-inflammatory signaling across diverse stress models, which may help maintain epithelial homeostasis (Wei et al., 2025; Ye et al., 2023). Quercetin has also been reported to exhibit antiviral activities against certain viruses (Di Petrillo et al., 2022; Gong et al., 2024), although the underlying mechanisms remain to be clarified. Notably, accumulating evidence suggests that quercetin can alleviate virus infection-associated intestinal injury (Feng et al., 2024; Wang et al., 2025). However, the physiological functions of quercetin in porcine intestinal epithelial cells are still poorly understood. More importantly, its regulatory role in a piglet RV infection model has not been systematically investigated.

Therefore, an RV challenge model in weaned pigs and an in vitro porcine intestinal epithelial cell system were used to evaluate the protective effects of dietary quercetin against RV-induced intestinal injury and to investigate potential mechanisms involving inflammatory responses, epithelial apoptosis, and barrier function. The results indicated that quercetin attenuated RV-induced intestinal inflammation and epithelial damage, supporting its potential as a nutritional strategy to mitigate RV-induced intestinal injury in weaned pigs.

## 2 Materials and methods

### 2.1 Animal ethics statement

The animal study protocol was reviewed and approved by the Institutional Animal Care and Use Committee of Sichuan Agricultural University (Approval No. SICAU20250153; approved on January 24, 2025). All procedures were carried out in the animal research facilities of Sichuan Agricultural University in accordance with institutional guidelines to ensure ethical treatment and welfare of the animals.

### 2.2 Quercetin source

The quercetin used in both the animal study and the cell-based assays was obtained from Guilin Layn Natural Ingredients Corp., Ltd. (Guilin, Guangxi, China) and was of 95% purity.

### 2.3 Virus, animals, experimental design, and diets

Porcine rotavirus OSU strain (ATCC VR-893) was used in the present study. The virus was serially passaged five times in IPEC-J2 cells, and the fifth-passage viral suspension was used to prepare a single working virus stock. Aliquots of the working stock were stored at −80 °C and used for all RV challenges in piglets. The infectious titer of the working inoculum was determined in MA104 and IPEC-J2 cells by the 50% tissue culture infectious dose (TCID_50_) assay (Reed and Muench, 1938). The titers were 10^7^ TCID_50_/mL in MA104 cells and 10^6.5^ TCID_50_/mL in IPEC-J2 cells. To confirm the identity of the working virus stock, the *VP4* and *VP7* genes were sequenced and compared with the reference porcine rotavirus OSU strain. Detailed sequence alignment results are provided in Table S1 and Fig. S1.

A total of 32 weaned pigs (Duroc × Landrace × Yorkshire) at 21 d of age, with an initial body weight of 6.49 ± 0.04 kg, were randomly allocated to four dietary treatments (*n* = 8 per group). The groups included a non-challenged control (CON group) receiving a basal diet, an RV-challenged group fed the basal diet (RV group), and two quercetin-supplemented groups challenged with RV that received the basal diet containing either 100 (RV + 100QUE group) or 200 mg/kg quercetin (RV + 200QUE group). The feeding trial lasted 17 d. Rotavirus challenge was performed from d 15 to 17 of the trial (3 consecutive days). Piglets in the RV, RV  +  100QUE, and RV  +  200QUE groups were orally gavaged with 25 mL of the RV suspension, whereas CON piglets received an equal volume of DMEM/F12 medium.

The basal diet (Table 1) was formulated to meet the nutrient requirements recommended by NRC (2012). Piglets were housed individually in metabolism cages (1.5 m × 0.7 m) with ad libitum access to feed and fresh water. The animal room was maintained at 25 to 28 °C with relative humidity of (65 ± 5)%. The quercetin concentrations in the experimental diets were determined by high-performance liquid chromatography with ultraviolet detection (HPLC-UV) using an Agilent 1260 Infinity II HPLC system (Agilent Technologies, Inc., Santa Clara, CA, USA), with reference to a previously reported feed-based analytical procedure (National Toxicology Program, 1992). Representative chromatograms are shown in Fig. S2.

**Table 1:** Ingredients and nutrient levels of experimental diets (as fed basis, %).

Ingredients	Contents	Nutrients	Contents
Corn	30.05	**Calculated nutrient levels**^2^	
Extruded corn	21.90	Digestible energy, MJ/kg	14.69
Soybean meal	6.50	Crude protein	19.68
Extruded full-fat soybean	12.50	Calcium	0.80
Fish meal	4.10	Available phosphorus	0.37
Whey powder	10.50	Lysine	1.40
Soybean protein concentrate	8.00	Methionine	0.45
Soybean oil	1.50	Methionine + cysteine	0.62
Sucrose	2.00	Threonine	0.81
Limestone	1.00	Tryptophan	0.21
NaCl	0.25	**Analyzed nutrient levels**	
Dicalcium phosphate	0.50	Crude protein	19.56
L-Lysine HCl (78%)	0.50	Gross energy, MJ/kg	18.20
DL-Methionine	0.20	Ether extract	3.33
L-Threonine (98.5%)	0.13		
Tryptophan (98%)	0.03		
Choline chloride	0.10		
Vitamin and mineral premix^1^	0.24		
Total	100.00		

1 The vitamin premix provided per kg of the diet: VA9000 IU; VD_3_ 3000 IU; VE 20 IU; VK_3_ 3 mg; VB_1_ 1.5 mg; VB_2_ 4 mg; VB_6_ 3 mg; VB_12_ 0.02 mg; niacin 30 mg; pantothenic acid 15 mg; folic acid 0.75 mg; and biotin 0.1 mg. The mineral premix provided per kg of the diet: Fe (FeSO_4_·H_2_O) 100 mg; Cu (CuSO_4_·5H_2_O) 6 mg; Zn (ZnSO_4_·H_2_O) 100 mg; Mn (MnSO_4_·H_2_O) 4 mg; I (KI) 0.3 mg; and Se (Na_2_SeO_3_) 0.3 mg.

2 The calculated nutrient levels of the diet were obtained from the China Feed Database (2020).

### 2.4 Chemical analysis and calculation

The crude protein (CP) content of the diet was analyzed with an automatic Kjeldahl nitrogen analyzer (K1100, Jinan Hanon Instruments Co., Ltd., Jinan, Shandong, China) in accordance with method 976.05 (AOAC, 2007). Ether extract (EE) was quantified using a Soxhlet extraction system (SN–00051B, Chengdu Shuniu Technology Co., Ltd., Chengdu, Sichuan, China) based on method 922.06 (AOAC, 2007). Gross energy (GE) was determined with an automated oxygen bomb calorimeter (Model 6400, Parr Instrument Company, Moline, IL, USA) according to ISO 9831:1998 (ISO, 1998).

The calculated values for digestible energy, crude protein, calcium, available phosphorus, lysine, methionine, methionine + cysteine, threonine, and tryptophan were obtained based on the China Feed Database (2020).

### 2.5 Sample collection and processing

On d 17, blood was drawn from the jugular vein into two separate 10 mL evacuated tubes. One tube was used for hematological measurements. The other tube was centrifuged at 3500 × *g* to harvest serum, which was stored at −20 °C until biochemical analysis. At the end of the trial, piglets were anesthetized by intravenous administration of sodium pentobarbital, and the duodenum, jejunum, and ileum were excised for sampling. A 4-cm mid-segment from each intestinal region was gently rinsed with ice-cold phosphate-buffered saline and fixed in 4% paraformaldehyde for morphological evaluation. In addition, mucosal tissue was scraped from the remaining intestinal segments, transferred to cryovials, snap-frozen in liquid nitrogen, and stored at −80 °C until further analysis.

To assess apoptosis in jejunal epithelial cells, single-cell suspensions were prepared from jejunal mucosa. Fresh jejunal mucosa was collected, minced, and incubated in 10 mL Hanks' Balanced Salt Solution supplemented with dithiothreitol at 37 °C for 30 min to remove mucus. Tissue fragments were washed and then incubated in 10 mL Hanks’ balanced salt solution (HBSS) containing 0.5 mmol/L ethylenediaminetetraacetic acid (EDTA) at 37 °C for 30 min with shaking to release epithelial cells. The EDTA supernatant was collected, and this step was repeated as needed. Collected fractions were pooled, filtered through a 70 μm strainer, and centrifuged to obtain epithelial cells for immediate flow cytometric analysis.

### 2.6 Growth performance

Feed intake was recorded daily for each piglet. Body weights were measured after a 12 h fast on d 0, 14, and 17, and these data were used to determine average daily feed intake (ADFI), average daily gain (ADG), and the feed-to-gain ratio (F/G).

### 2.7 Blood parameters analysis

Serum biochemical parameters were measured using an automatic biochemical analyzer (Model 3100, Hitachi High-Tech Corporation, Tokyo, Japan) with commercial assay kits from Maccura Biotechnology Co., Ltd. (Chengdu, Sichuan, China), including total protein (TP; Cat. No. CH0101008), albumin (ALB; Cat. No. CH0101002), alkaline phosphatase (ALP; Cat. No. CH0101203), urea (UREA; Cat. No. CH0101051), creatinine (CREA; Cat. No. CH0101053), alanine aminotransferase (ALT; Cat. No. CH0101201), aspartate aminotransferase (AST; Cat. No. CH0101202), glucose (GLU; Cat. No. CH0101102), total cholesterol (TC; Cat. No. CH0101152), triglycerides (TG; Cat. No. CH0101151), high-density lipoprotein cholesterol (HDL-C; Cat. No. CH0101161), and low-density lipoprotein cholesterol (LDL-C; Cat. No. CH0103162). Globulin (GLOB) concentration was calculated as the difference between TP and ALB.

Hematological parameters were determined in fresh anticoagulated whole blood using an automated veterinary hematology analyzer (BC-5000 Vet, Shenzhen Mindray Bio-Medical Electronics Co., Ltd., Shenzhen, Guangdong, China) with the manufacturer-recommended reagents, including V-52 D Diluent, V-52 DIFF Lyse, V-52 LH Lyse, and Probe Cleanser, according to the manufacturer's instructions. The measured parameters included white blood cell count (WBC), absolute neutrophil count (Neu#), absolute lymphocyte count (Lym#), absolute monocyte count (Mon#), absolute eosinophil count (Eos#), absolute basophil count (Bas#), red blood cell count (RBC), hemoglobin (HGB), hematocrit (HCT), mean corpuscular volume (MCV), mean corpuscular hemoglobin (MCH), mean corpuscular hemoglobin concentration (MCHC), red cell distribution width-coefficient of variation (RDW-CV), red cell distribution width-standard deviation (RDW-SD), platelet count (PLT), mean platelet volume (MPV), platelet distribution width (PDW), and plateletcrit (PCT).

Serum concentrations of interleukin-1β (IL-1β; Cat. No. RX501070P), tumor necrosis factor-α (TNF-α; Cat. No. RX500888P), interleukin-6 (IL-6; Cat. No. RX501064P), interleukin-10 (IL-10; Cat. No. RX501078P), and D-lactate (Cat. No. RXJ501159P) were analyzed using enzyme-linked immunosorbent assay (ELISA) kits from Quanzhou Ruixin Biological Technology Co., Ltd. (Quanzhou, Fujian, China) according to the manufacturer's instructions. Absorbance was measured using a microplate reader (SpectraMax M2, Molecular Devices, LLC, San Jose, CA, USA). Serum diamine oxidase (DAO) activity was analyzed using a commercial assay kit (Cat. No. A088-3-1, Nanjing Jiancheng Bioengineering Institute, Nanjing, Jiangsu, China) according to the manufacturer's instructions, with absorbance measured using the same microplate reader.

### 2.8 Intestinal morphology analysis

For intestinal histomorphology, duodenal, jejunal, and ileal segments were collected and fixed in 4% paraformaldehyde. After trimming, tissues were dehydrated through graded ethanol, embedded in paraffin, and sectioned at 5 μm using a rotary microtome (PM-24, Wuhan Servicebio Technology Co., Ltd., Wuhan, Hubei, China). Sections were deparaffinized in xylene and rehydrated through descending alcohol concentrations, followed by hematoxylin and eosin staining. Slides were examined by light microscopy (BX43FC, Olympus Corporation, Tokyo, Japan), and morphometric measurements were performed on 10 well-oriented fields per sample with clearly defined villus-crypt units. Villus height and crypt depth were quantified using Image-Pro Plus 6.0 (Media Cybernetics, Inc., Rockville, MD, USA), and the villus-to-crypt ratio (V:C) was calculated.

### 2.9 Determination of the enzyme activities of the intestinal mucosa

After thawing, intestinal mucosal samples were homogenized in sterile normal saline at a ratio of 1 g tissue to 9 mL saline. The homogenates were centrifuged at 3500 × *g* for 15 min at 4 °C, and the supernatants were collected and stored at −80 °C until analysis. Disaccharidase activities were determined using commercial assay kits, including the lactase activity assay kit (Cat. No. E-BC-K131-M), maltase activity assay kit (Cat. No. E-BC-K041-M), and sucrase activity assay kit (Cat. No. E-BC-K751-M), all purchased from Elabscience Biotechnology Co., Ltd. (Wuhan, Hubei, China), according to the manufacturer's instructions. Absorbance was measured at 505 nm using a microplate reader (SpectraMax M2, Molecular Devices, LLC, San Jose, CA, USA).

### 2.10 RNA extraction and gene expression analysis

Total RNA from intestinal tissues or cultured cells was isolated using RNAiso Plus (Cat. No. 9108, Takara Biotechnology (Dalian) Co., Ltd., Dalian, Liaoning, China) according to the manufacturer's instructions. RNA concentration and purity were determined using a NanoDrop One spectrophotometer (Thermo Fisher Scientific Inc., Waltham, MA, USA). Reverse transcription was performed using 1 μg of total RNA with PrimeScript RT Master Mix (Cat. No. RR036A, Takara Biotechnology (Dalian) Co., Ltd., Dalian, Liaoning, China) to synthesize complementary DNA (cDNA). Quantitative real-time PCR was performed using TB Green Premix Ex Taq II (Cat. No. RR820A, Takara Biotechnology (Dalian) Co., Ltd., Dalian, Liaoning, China) on a QuantStudio 5 Real-Time PCR System (Thermo Fisher Scientific Inc., Waltham, MA, USA). Reactions were performed in a final volume of 10 μL containing 5 μL TB Green Premix Ex Taq II (Cat. No. RR820A, Takara Biotechnology (Dalian) Co., Ltd., Dalian, Liaoning, China), 0.4 μL each of forward and reverse primers (10 μmol/L), 1 μL cDNA, and 3.2 μL nuclease-free water. Amplification conditions consisted of 95 °C for 30 s, followed by 40 cycles of 95 °C for 5 s and 60 °C for 30 s. β-Actin was used as the internal reference gene, and relative transcript abundance was calculated using the 2^−ΔΔCt^ method (Livak and Schmittgen, 2001). Primers were designed, where possible, to span exon–exon junctions and were synthesized by Sangon Biotech (Shanghai) Co., Ltd. (Shanghai, China). The full names of the abbreviations of all genes can be found in supplementary file abbreviations and full names of genes. The primer sequences are listed in Table S2.

### 2.11 Immunohistochemistry analysis of the secretory immunoglobulin A (sIgA)-positive cells

Intestinal tissue samples were fixed in 4% formaldehyde, paraffin-embedded, and cut into 4-μm sections. For optimal antigen exposure, heat-induced epitope retrieval was performed using microwave heating in sodium citrate buffer (pH 6.0). After three rinses with phosphate-buffered saline (PBS), endogenous peroxidase activity was quenched with 3% H_2_O_2_. Sections were then blocked with 10% normal goat serum for 30 min to minimize nonspecific binding. The sections were incubated overnight at 4 °C with an anti-sIgA primary antibody diluted in bovine serum albumin solution, followed by incubation with a biotin-conjugated secondary antibody for 1 h at room temperature. Immunoreactivity was visualized using a 3,3′-diaminobenzidine (DAB) substrate, and nuclei were counterstained with hematoxylin. Slides were dehydrated through graded ethanol, cleared in xylene, and mounted with neutral resin for permanent preservation. Digital images were acquired using a whole-slide scanner (LG-S80, Wuhan Servicebio Technology Co., Ltd., Wuhan, Hubei, China) and viewed using Saiviewer digital slide viewing software (Wuhan Servicebio Technology Co., Ltd., Wuhan, Hubei, China).

### 2.12 Immunofluorescence analysis of the tight junction proteins

Intestinal specimens were fixed in 4% paraformaldehyde for 24 h and then cryoprotected by immersion in 30% sucrose at 4 °C. Tissues were cut into 5 μm cryosections using a temperature-controlled cryostat (CryoStar NX50, Epredia Laboratory Products Manufacturing (Shanghai) Co., Ltd., Shanghai, China) set at −20 °C. After antigen retrieval, sections were permeabilized with 0.5% Triton X-100 and blocked with 10% normal goat serum. For immunofluorescence, sections were incubated overnight at 4 °C with an anti-ZO-1 primary antibody (1:100) in a humidified chamber. After PBS washes, fluorophore-conjugated secondary antibodies were applied for 60 min at room temperature in the dark. Nuclei were counterstained with 4′,6-diamidino-2-phenylindole (DAPI), and slides were mounted using an anti-fade medium. Fluorescence images were acquired using a confocal laser-scanning microscope (FV1000, Olympus Corporation, Tokyo, Japan) with appropriate filter settings.

### 2.13 Flow cytometric analysis of apoptosis

Apoptosis was assessed by flow cytometry using a FITC Annexin V Apoptosis Detection Kit I (Cat. No. 556547, Becton, Dickinson and Company, San Jose, CA, USA) with different membrane-impermeant viability dyes depending on the sample type. For jejunal epithelial cells, freshly prepared single-cell suspensions were washed twice with ice-cold PBS and resuspended in 1 × binding buffer, followed by staining with fluorochrome-conjugated Annexin V and DAPI for 15 min at room temperature in the dark. For IPEC-J2 cells, after the indicated treatments, cells were collected by gentle trypsinization, washed, resuspended in 1 × binding buffer, and stained with fluorochrome-conjugated Annexin V and propidium iodide (PI) under the same conditions. Samples were acquired within 1 h using a CytoFLEX S flow cytometer (Beckman Coulter, Inc., Brea, CA, USA).

### 2.14 Cell culture and treatments

The porcine intestinal epithelial cell line IPEC-J2 was obtained from our laboratory cell bank and maintained in Dulbecco's Modified Eagle Medium/Nutrient Mixture F-12 (DMEM/F-12; Cat. No. 11320033, Gibco, Thermo Fisher Scientific Inc., Waltham, MA, USA) supplemented with 10% fetal bovine serum (FBS; Cat. No. 10099141C, Gibco, Thermo Fisher Scientific Inc., Waltham, MA, USA) and 1% penicillin-streptomycin (Cat. No. 15140122, Gibco, Thermo Fisher Scientific Inc., Waltham, MA, USA). Cells were cultured at 37 °C in a humidified incubator containing 5% CO_2_ and routinely passaged at 70%–80% confluence.

Quercetin was dissolved in dimethyl sulfoxide (DMSO) to prepare a stock solution and diluted in complete medium to the indicated final concentrations. For infection experiments, IPEC-J2 cells were pretreated with quercetin for 1 h, and quercetin was maintained during the subsequent 24 h RV infection at a multiplicity of infection (MOI) of 10. Control groups received the same final concentration of DMSO. After infection, cells were harvested for downstream assays as specified, including viability measurement, gene expression analysis, apoptosis assessment, and immunofluorescence staining.

### 2.15 Cell viability assay

For viability measurements, IPEC-J2 cells were seeded into 96-well plates and allowed to adhere overnight. Cells were then subjected to the indicated treatments, including quercetin pretreatment for 1 h and RV infection (MOI = 10) for 24 h. After treatment, 10 μL of Cell Counting Kit-8 (CCK-8) reagent (Cat. No. C0038, Beyotime Biotechnology Co., Ltd., Shanghai, China) was added to each well, followed by incubation at 37 °C for 1 h in the dark. Absorbance was read at 450 nm using a microplate reader (SpectraMax M2, Molecular Devices, LLC, San Jose, CA, USA). Cell viability was expressed relative to the untreated control, with the untreated control normalized to 1.

### 2.16 Cell immunofluorescence staining

For ZO-1 staining, IPEC-J2 cells grown in glass-bottom confocal dishes were gently washed once with PBS and fixed in situ with ice-cold methanol at 4 °C for 10 min. After three PBS washes, cells were blocked with 3% bovine serum albumin (BSA) for 30 min at room temperature, incubated with an anti-ZO-1 primary antibody for 1 h, and then incubated with the corresponding fluorophore-conjugated secondary antibody for 1 h at room temperature in the dark, with three PBS washes between steps. For NF-κB p65 staining, cells were washed once with PBS, fixed in 4% paraformaldehyde at room temperature for 15 min, and washed three times with PBS. Cells were permeabilized with 0.05% Triton X-100 for 10 min, blocked with 3% BSA for 30 min, and incubated with an anti–NF–κB p65 primary antibody (1:500; diluted in 0.05% Triton X-100/PBS) for 1 h at room temperature. After PBS washes, cells were incubated with the appropriate fluorophore-conjugated secondary antibody for 1 h at room temperature in the dark. For both assays, samples were mounted using 50 μL anti-fade mounting medium containing DAPI and stored at 4 °C protected from light until imaging. Images were acquired using a FLUOVIEW FV4000 confocal laser-scanning microscope (Evident Corporation, Tokyo, Japan).

### 2.17 Public transcriptomic data analysis

Transcriptomic dataset GSE300313 was downloaded from the NCBI Gene Expression Omnibus (GEO) database. This dataset comprises bulk RNA-Seq profiles of the human intestinal cell line T84. Gene expression was quantified using raw read counts. Differentially expressed genes (DEG) were identified using DESeq2 with the criteria of |fold change| > 2 and *P* < 0.05. Gene Ontology (GO) enrichment, Kyoto Encyclopedia of Genes and Genomes (KEGG) pathway enrichment, and gene set enrichment analysis (GSEA) were performed using the OmicShare online platform (accessed January 5, 2026).

### 2.18 Statistical analysis

Data were processed using Excel 2019, and statistical analyses were performed using SPSS 27.0 (IBM Corp., Armonk, NY, USA). Homogeneity of variances across treatments was assessed using Levene's test. Data were analyzed by one-way analysis of variance. When a significant main effect was detected, multiple comparisons were conducted using Tukey's test. The statistical model used for data analysis was:

$$Y_{ij}=\mu +T_{i}+\varepsilon_{ij}\text{,}$$

where *Y*_*ij*_ is the observed value of the *j*-th pig in the *i*-th treatment group, μ is the overall mean, *T*_*i*_ is the fixed effect of treatment (*i* = CON, RV, RV + 100QUE, and RV + 200QUE), and *ε*_*ij*_ is the random residual error. In addition, linear and quadratic polynomial contrasts were performed among RV-infected piglets receiving 0, 100, or 200 mg/kg quercetin. For the equally spaced quercetin levels, the contrast coefficients were −1, 0, and 1 for the linear contrast, and 1, −2, and 1 for the quadratic contrast. All experimental data are presented as means and standard error of the mean (SEM). Differences were considered statistically significant at *P* < 0.05, while values of 0.05 ≤ *P* < 0.10 were considered to indicate a trend toward significance.

## 3 Results

### 3.1 Effects of dietary quercetin supplementation on growth performance of weaned pigs challenged with RV

Growth performance during the pre-challenge (d 0–14) and post–challenge (d 15–17) periods, including ADFI, ADG, and F/G, is summarized in Table 2. During d 0 to 14, ADFI, ADG, and F/G did not differ among treatments (*P* > 0.05). During d 15 to 17, RV challenge increased F/G compared with the CON group (*P* = 0.017). Within RV-challenged piglets, dietary supplementation with 200 mg/kg quercetin reduced F/G compared with the RV group (*P* = 0.017), whereas dietary supplementation with 100 mg/kg quercetin did not differ from the RV group (*P* > 0.05). A significant linear decreasing trend in F/G was detected with increasing quercetin dose within RV-challenged pigs (linear *P* = 0.002). No differences were observed in ADFI or ADG among treatments during d 15 to 17 (*P* > 0.05).

**Table 2:** Effects of dietary quercetin (QUE) supplementation on growth performance of weaned pigs before and after rotavirus (RV) challenge.

Item	Treatments^1^				SEM	*P-*value^2^		
	CON	RV	RV + 100 QUE	RV + 200 QUE		ANOVA	Linear	Quadratic
**0**–**14 d (Pre-challenge)**								
ADFI, g	310.6	316.2	309.1	306.2	13.64	0.996	0.798	0.949
ADG, g	209.2	206.1	200.5	198.0	9.55	0.933	0.793	0.951
F/G	1.48	1.53	1.54	1.55	0.067	0.757	0.512	0.594
**15**–**17 d (Post–challenge)**								
ADFI, g	427.2	545.3	471.7	467.7	24.58	0.316	0.206	0.482
ADG, g	325.0	337.3	331.2	348.6	21.37	0.985	0.960	0.831
F/G	1.31^b^	1.62^a^	1.42^ab^	1.34^b^	0.079	0.017	0.002	0.871

ADG = average daily gain; ADFI = average daily feed intake; F/G = feed-to-gain ratio; SEM = standard error of the mean.

Values in the same row with different superscript letters differ significantly (*P* < 0.05); *n* = 8.

1 CON, non-challenged pigs fed the basal diet; RV, RV-challenged pigs fed the basal diet; RV  +  100QUE, RV-challenged pigs fed the basal diet supplemented with 100 mg/kg QUE; RV  +  200QUE, RV-challenged pigs fed the basal diet supplemented with 200 mg/kg QUE.

2 The ANOVA *P*-value represents the overall comparison among all four treatment groups. Linear and quadratic *P*-values were obtained from polynomial contrasts among RV-challenged pigs receiving 0, 100, or 200 mg/kg QUE.

### 3.2 Effects of dietary quercetin supplementation on systemic biochemical and hematological parameters of weaned pigs challenged with RV

A selection of representative systemic biochemical and hematological parameters is presented in Table 3 (complete serum biochemical parameters are provided in Table S3 and complete hematological parameters are presented in Table S4). Relative to the CON group, RV challenge decreased ALB (*P* = 0.034) and increased ALP (*P* = 0.007). Within RV-challenged piglets, dietary supplementation with 100 and 200 mg/kg quercetin increased ALB compared with the RV group (*P* = 0.034), with a significant linear increasing trend as quercetin dose increased (linear *P* = 0.016). Under RV challenge, dietary supplementation with 200 mg/kg quercetin reduced ALP compared with the RV group (*P* = 0.007), and ALP showed a significant linear decreasing trend with increasing quercetin dose (linear *P* = 0.014). In addition, UREA showed a significant linear increasing trend with increasing quercetin dose within RV-challenged piglets (linear *P* = 0.038). No significant treatment effects were observed for the remaining serum biochemical parameters, including CREA, GLU, TC, TG, HDL-C, and LDL-C (*P* > 0.05; Table S3).

**Table 3:** Effects of dietary quercetin (QUE) on serum biochemical indices and hematological parameters in weaned pigs challenged with rotavirus (RV).

Item	Treatments^1^				SEM	*P-*value^2^		
	CON	RV	RV + 100 QUE	RV + 200 QUE		ANOVA	Linear	Quadratic
**Nutritional status and protein metabolism**								
TP, g/L	50.85^a^	46.91^b^	52.90^a^	50.48^ab^	0.878	0.042	0.096	0.086
ALB, g/L	25.30^a^	22.08^b^	24.92^a^	25.01^a^	0.457	0.034	0.016	0.171
GLOB, g/L	25.55	24.83	27.98	25.47	0.714	0.071	0.405	0.121
UREA, mmol/L	4.55	3.00	4.08	4.30	0.229	0.065	0.038	0.432
**Systemic stress-related enzyme, U/L**								
ALP	199.07^b^	287.76^a^	257.14^ab^	209.24^b^	10.171	0.007	0.014	0.679
AST	65.84	74.70	77.31	69.11	3.057	0.440	0.442	0.413
ALT	81.63	67.38	88.26	84.57	3.295	0.119	0.069	0.129
**Hematological parameters**								
RBC, × 10^12^/L	5.94^a^	4.77^b^	6.04^a^	5.83^a^	0.154	0.007	0.021	0.055
HGB, g/L	115.71^a^	90.10^b^	112.25^a^	111.38^a^	3.136	0.012	0.023	0.123
HCT, %	33.96^a^	27.24^b^	32.93^a^	32.88^a^	0.892	0.029	0.040	0.192
PLT, × 10^9^/L	351.83^b^	514.48^a^	444.33^ab^	359.86^b^	31.203	0.025	0.105	0.930

TP = total protein; ALB = albumin; GLOB = globulin; UREA = urea; ALP = alkaline phosphatase; ALT = alanine aminotransferase; RBC = redblood cell count; HGB = hemoglobin; HCT = hematocrit; PLT = platelet count; SEM = standard error of the mean.

Values in the same row with different superscript letters differ significantly (*P* < 0.05); *n* = 8.

1 CON, non-challenged pigs fed the basal diet; RV, RV-challenged pigs fed the basal diet; RV  +  100QUE, RV-challenged pigs fed the basal diet supplemented with 100 mg/kg QUE; RV  +  200QUE, RV-challenged pigs fed the basal diet supplemented with 200 mg/kg QUE.

2 The ANOVA *P*-value represents the overall comparison among all four treatment groups. Linear and quadratic *P*-values were obtained from polynomial contrasts among RV-challenged pigs receiving 0, 100, or 200 mg/kg QUE.

For hematological parameters, RV challenge decreased RBC, HGB, and HCT and increased PLT compared with the CON group (*P* < 0.05). Within RV-challenged piglets, dietary supplementation with 100 and 200 mg/kg quercetin significantly alleviated the RV-induced decreases in RBC, HGB, and HCT compared with the RV group (*P* < 0.05), and significant linear increasing trends were detected as quercetin dose increased (linear *P* = 0.021, *P* = 0.023, and *P* = 0.040). No significant treatment effects were detected for the remaining hematological parameters (*P* > 0.05; Table S4), although eosinophil count and mean corpuscular hemoglobin concentration tended to differ among treatments (*P* = 0.074 and *P* = 0.051).

### 3.3 Effects of dietary quercetin supplementation on intestinal and systemic inflammatory responses of weaned pigs challenged with RV

Serum and jejunal inflammatory cytokines are presented in Table 4. Compared with the CON group, RV challenge increased serum TNF-α, IL-1β, and IL-6 contents and decreased IL-10 content (*P* < 0.05). Moreover, serum TNF-α and IL-6 contents showed significant linear decreasing trends with increasing quercetin dose (linear *P* < 0.001 and *P* = 0.002), whereas IL-10 content showed a significant linear increasing trend (linear *P* < 0.001). In the jejunum, RV challenge increased TNF-α, IL-1β, and IL-6 contents and decreased IL-10 content compared with the CON group (*P* < 0.05). Within RV-challenged pigs, dietary supplementation with 200 mg/kg quercetin decreased jejunal TNF-α and IL-6 contents and increased IL-10 content compared with the RV group (*P* < 0.05). In addition, under RV challenge, jejunal TNF-α, IL-6, and IL-1β contents exhibited significant linear decreasing trends with increasing quercetin dose (linear *P* < 0.001, *P* < 0.001, and *P* = 0.021), whereas IL-10 content showed a significant linear increasing trend (linear *P* < 0.001). Notably, IL-1β content also showed a significant quadratic response to quercetin supplementation (quadratic *P* = 0.038), indicating a potential non-linear dose-related pattern.

**Table 4:** Effects of dietary quercetin (QUE) supplementation on inflammatory cytokine concentrations in serum and jejunum of weaned pigs challenged with rotavirus (RV).

Item	Treatment^1^				SEM	*P-*value^2^		
	CON	RV	RV + 100 QUE	RV + 200 QUE		ANOVA	Linear	Quadratic
**Serum inflammatory cytokines**, **pg/mL**								
TNF-α	61.15^c^	115.76^a^	95.45^ab^	76.81^bc^	4.800	<0.001	<0.001	0.931
IL-1β	341.11^b^	438.47^a^	425.03^a^	382.50^ab^	12.556	0.019	0.072	0.566
IL-6	381.82^b^	629.44^a^	577.68^a^	437.15^b^	21.828	<0.001	0.002	0.121
IL-10	136.33^a^	89.51^c^	109.22^bc^	120.86^ab^	3.896	<0.001	<0.001	0.464
**Jejunal inflammatory cytokines, pg/g**								
TNF-α	537.89^c^	1076.27^a^	868.60^ab^	713.80^bc^	53.223	<0.001	<0.001	0.778
IL-1β	2528.15^c^	4403.01^ab^	4642.03^a^	3511.78^b^	188.425	<0.001	0.021	0.038
IL-6	3437.65^c^	5927.12^a^	4925.83^ab^	3960.16^bc^	223.598	<0.001	<0.001	0.963
IL-10	1358.13^a^	743.21^c^	930.13^c^	1145.35^b^	48.700	<0.001	<0.001	0.811

TNF-α  =  tumor necrosis factor-α; IL-1β = interleukin-1β; IL-6  =  interleukin-6; IL-10  =  interleukin-10; SEM  =  standard error of the mean.

Values in the same row with different superscript letters differ significantly (*P* < 0.05); *n* = 8.

1 CON, non-challenged pigs fed the basal diet; RV, RV-challenged pigs fed the basal diet; RV  +  100QUE, RV-challenged pigs fed the basal diet supplemented with 100 mg/kg QUE; RV  +  200QUE, RV-challenged pigs fed the basal diet supplemented with 200 mg/kg QUE.

2 The ANOVA *P*-value represents the overall comparison among all four treatment groups. Linear and quadratic *P*-values were obtained from polynomial contrasts among RV-challenged pigs receiving 0, 100, or 200 mg/kg QUE.

### 3.4 Effects of dietary quercetin supplementation on intestinal morphology and jejunal RV-VP6 fluorescence in weaned pigs challenged with RV

Intestinal morphology in the duodenum, jejunum, and ileum is presented in Table 5 and Fig. 1A. Compared with the CON group, RV challenge decreased duodenal villus height and V:C (*P* < 0.05). Within RV-challenged pigs, duodenal V:C showed a significant linear increasing trend with increasing quercetin dose (linear *P* = 0.007). In the jejunum, RV challenge decreased villus height and V:C compared with the CON group (*P* < 0.05). Within RV-challenged piglets, dietary supplementation with 100 mg/kg quercetin increased jejunal villus height and V:C (*P* < 0.05), and dietary supplementation with 200 mg/kg quercetin increased V:C and villus height (*P* < 0.05) compared with the RV group. Moreover, jejunal villus height and V:C exhibited significant linear increasing trends with increasing quercetin dose (linear *P* = 0.012 and *P* = 0.013). In the ileum, RV challenge decreased villus height and V:C compared with the CON group (*P* < 0.05). Within RV-challenged piglets, dietary supplementation with 100 and 200 mg/kg quercetin increased ileal villus height and V:C (*P* < 0.05) compared with the RV group. In addition, under RV challenge, ileal villus height and V:C showed significant linear increasing trends with increasing quercetin dose (linear *P* < 0.001 and *P* < 0.001), while crypt depth showed a significant linear decreasing trend (linear *P* = 0.023). Ileal villus height also showed a significant quadratic response (quadratic *P* = 0.026). Representative immunofluorescence images of RV-VP6 in the jejunum are shown in Fig. 1B. Rotavirus challenge markedly increased RV-VP6-positive fluorescence signals in jejunal tissue, whereas dietary supplementation with 100 and 200 mg/kg quercetin reduced the relative RV-VP6 fluorescence intensity under RV challenge (*P* < 0.001).

**Table 5:** Effects of dietary quercetin (QUE) supplementation on small-intestinal morphology in weaned pigs challenged with rotavirus (RV).

Item	Treatments^1^				SEM	*P-*value^2^		
	CON	RV	RV + 100 QUE	RV + 200 QUE		ANOVA	Linear	Quadratic
**Duodenum**								
Villus height, μm	354.77^a^	274.40^b^	306.84^ab^	318.32^ab^	9.939	0.033	0.112	0.637
Crypt depth, μm	211.96	270.93	258.46	224.36	9.734	0.105	0.096	0.641
V:C	1.74^a^	1.03^b^	1.21^b^	1.48^ab^	0.073	0.001	0.007	0.757
**Jejunum**								
Villus height, μm	334.21^a^	221.09^b^	293.90^a^	327.66^a^	12.336	0.014	0.012	0.315
Crypt depth, μm	182.71	220.24	211.30	209.70	8.179	0.506	0.456	0.995
V:C	2.01^a^	1.03^c^	1.46^b^	1.57^ab^	0.107	0.028	0.013	0.270
**Ileum**								
Villus height, μm	299.46^a^	224.58^b^	285.67^a^	282.78^a^	7.839	<0.001	<0.001	0.026
Crypt depth, μm	178.33	215.00	192.46	171.16	6.399	0.069	0.023	0.971
V:C	1.68^a^	1.08^b^	1.55^a^	1.67^a^	0.063	<0.001	<0.001	0.193

V:C = villus height-to-crypt depth ratio; SEM = standard error of the mean.

Values in the same row with different superscript letters differ significantly (*P* < 0.05); *n* = 8.

1 CON, non-challenged pigs fed the basal diet; RV, RV-challenged pigs fed the basal diet; RV  +  100QUE, RV-challenged pigs fed the basal diet supplemented with 100 mg/kg QUE; RV  +  200QUE, RV-challenged pigs fed the basal diet supplemented with 200 mg/kg QUE.

2 The ANOVA *P*-value represents the overall comparison among all four treatment groups. Linear and quadratic *P*-values were obtained from polynomial contrasts among RV-challenged pigs receiving 0, 100, or 200 mg/kg QUE.

**Fig. 1:**
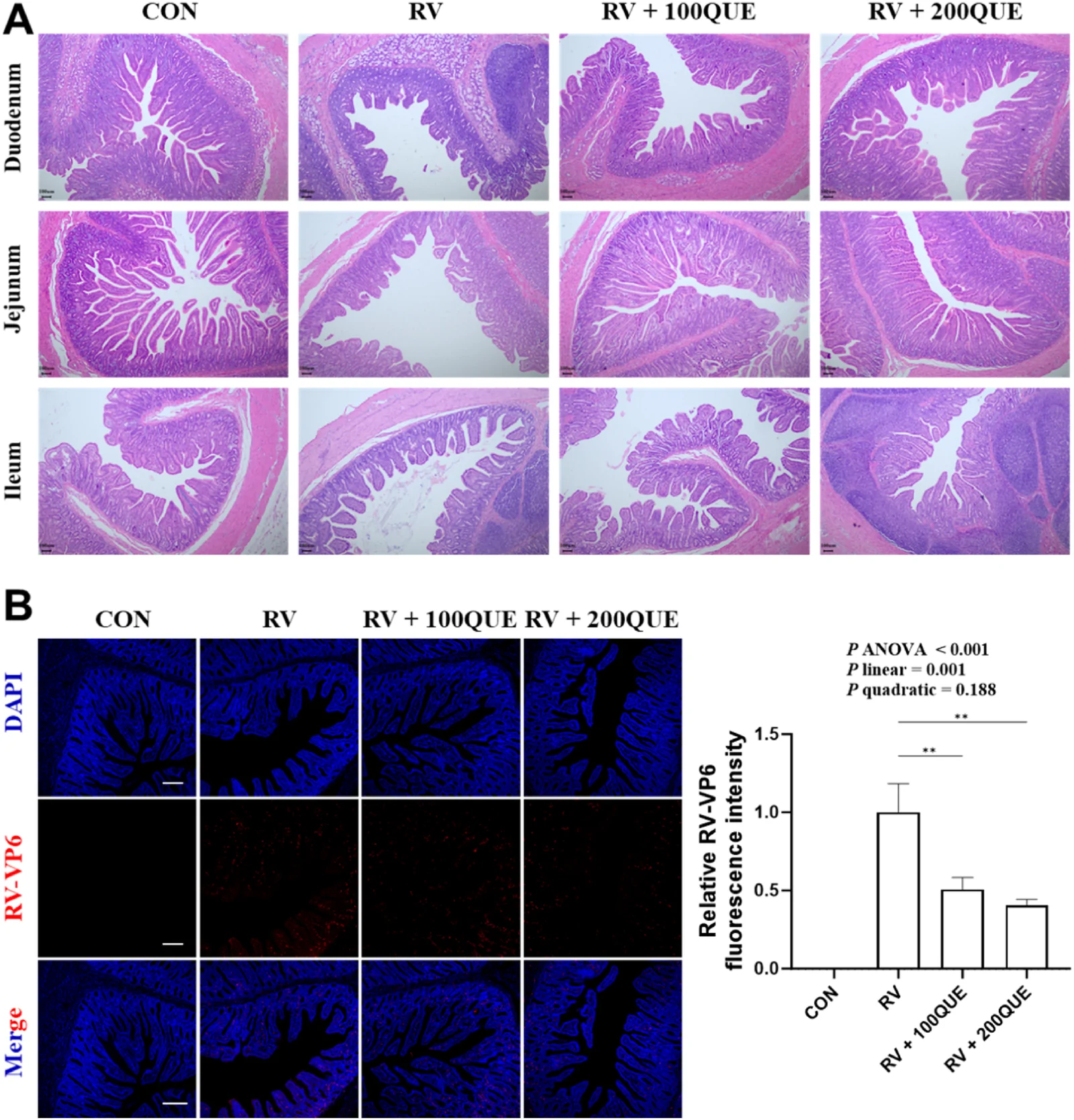
Dietary quercetin (QUE) supplementation improves small-intestinal histomorphology and reduces jejunal rotavirus (RV)-VP6 fluorescence in weaned pigs challenged with RV. (A) Representative hematoxylin and eosin (H&E)-stained sections of the duodenum, jejunum, and ileum. Scale bar = 100 μm. (B) Representative immunofluorescence images of RV-VP6 in jejunal sections. Nuclei were stained with 4′,6-diamidino-2-phenylindole (DAPI; blue), and RV-VP6-positive signals are shown in red. Scale bar = 100 μm. CON, non-challenged pigs fed the basal diet; RV, RV-challenged pigs fed the basal diet; RV  +  100QUE, RV-challenged pigs fed the basal diet supplemented with 100 mg/kg QUE; RV  +  200QUE, RV-challenged pigs fed the basal diet supplemented with 200 mg/kg QUE. The ANOVA *P*-value represents the overall comparison among all four treatment groups. Linear and quadratic *P*-values were obtained from polynomial contrasts among RV-challenged pigs receiving 0, 100, or 200 mg/kg QUE. Data are presented as mean ± standard error of the mean (SEM), *n* = 8. ∗∗*P* < 0.01.

### 3.5 Effects of dietary quercetin supplementation on intestinal digestive enzyme activities of weaned pigs challenged with RV

Disaccharidase activities in the duodenum, jejunum, and ileum are presented in Table 6. Compared with the CON group, RV challenge decreased duodenal lactase, maltase, and sucrase activities (*P* < 0.05). In the jejunum, RV challenge decreased maltase and sucrase activities (*P* < 0.05). In the ileum, RV challenge decreased lactase activity (*P* < 0.001). Within RV-challenged piglets, dietary supplementation with 200 mg/kg quercetin increased maltase activity in the duodenum and jejunum compared with the RV group (*P* = 0.011 and *P* = 0.017). Moreover, under RV challenge, duodenal maltase showed a significant linear increasing trend with increasing quercetin dose (linear *P* = 0.005), and jejunal lactase and maltase also exhibited significant linear increasing trends (linear *P* = 0.038 and *P* = 0.016). Ileal lactase also showed a significant quadratic response to quercetin supplementation (quadratic *P* = 0.048).

**Table 6:** Effects of dietary quercetin (QUE) supplementation on disaccharidase activities (U/mg prot) in the duodenum, jejunum, and ileum of weaned pigs challenged with rotavirus (RV).

Items	Treatments^1^				SEM	*P-*value^2^		
	CON	RV	RV + 100 QUE	RV + 200 QUE		ANOVA	Linear	Quadratic
**Duodenum**								
Lactase	18.19^a^	6.63^b^	8.32^b^	8.46^b^	1.361	0.003	0.394	0.673
Maltase	68.60^a^	35.78^b^	47.20^ab^	67.85^a^	4.490	0.011	0.005	0.597
Sucrase	29.79^a^	12.35^b^	14.37^b^	16.14^b^	1.956	0.001	0.080	0.945
**Jejunum**								
Lactase	36.81	18.42	21.23	33.15	3.098	0.089	0.038	0.430
Maltase	75.81^a^	42.41^b^	55.10^ab^	68.58^a^	4.655	0.017	0.016	0.953
Sucrase	41.36^a^	17.17^b^	24.81^ab^	31.95^ab^	3.304	0.049	0.070	0.967
**Ileum**								
Lactase	44.94^a^	25.95^b^	21.94^b^	33.68^ab^	2.438	<0.001	0.087	0.048
Maltase	64.46	54.51	51.89	73.34	3.810	0.172	0.027	0.091
Sucrase	48.09	33.38	27.75	41.58	3.150	0.099	0.241	0.115

SEM = standard error of the mean.

Values in the same row with different superscript letters differ significantly (*P* < 0.05); *n* = 8.

1 CON, non-challenged pigs fed the basal diet; RV, RV-challenged pigs fed the basal diet; RV  +  100QUE, RV-challenged pigs fed the basal diet supplemented with 100 mg/kg QUE; RV  +  200QUE, RV-challenged pigs fed the basal diet supplemented with 200 mg/kg QUE.

2 The ANOVA *P*-value represents the overall comparison among all four treatment groups. Linear and quadratic *P*-values were obtained from polynomial contrasts among RV-challenged pigs receiving 0, 100, or 200 mg/kg QUE.

### 3.6 Effects of dietary quercetin supplementation on jejunal nutrient transporter gene expression of weaned pigs challenged with RV

Jejunal nutrient transporter related mRNA expression is shown in Fig. 2. Compared with the CON group, RV challenge decreased relative *CAT1*, *SNAT2*, and *FATP1* mRNA expression (*P* < 0.05). Under RV challenge, dietary supplementation with 100 and 200 mg/kg quercetin increased relative *CAT1* mRNA expression compared with the RV group (*P* = 0.018), whereas dietary supplementation with 200 mg/kg quercetin also increased relative *SNAT2* and *FATP1* mRNA expression (*P* < 0.05).

**Fig. 2:**
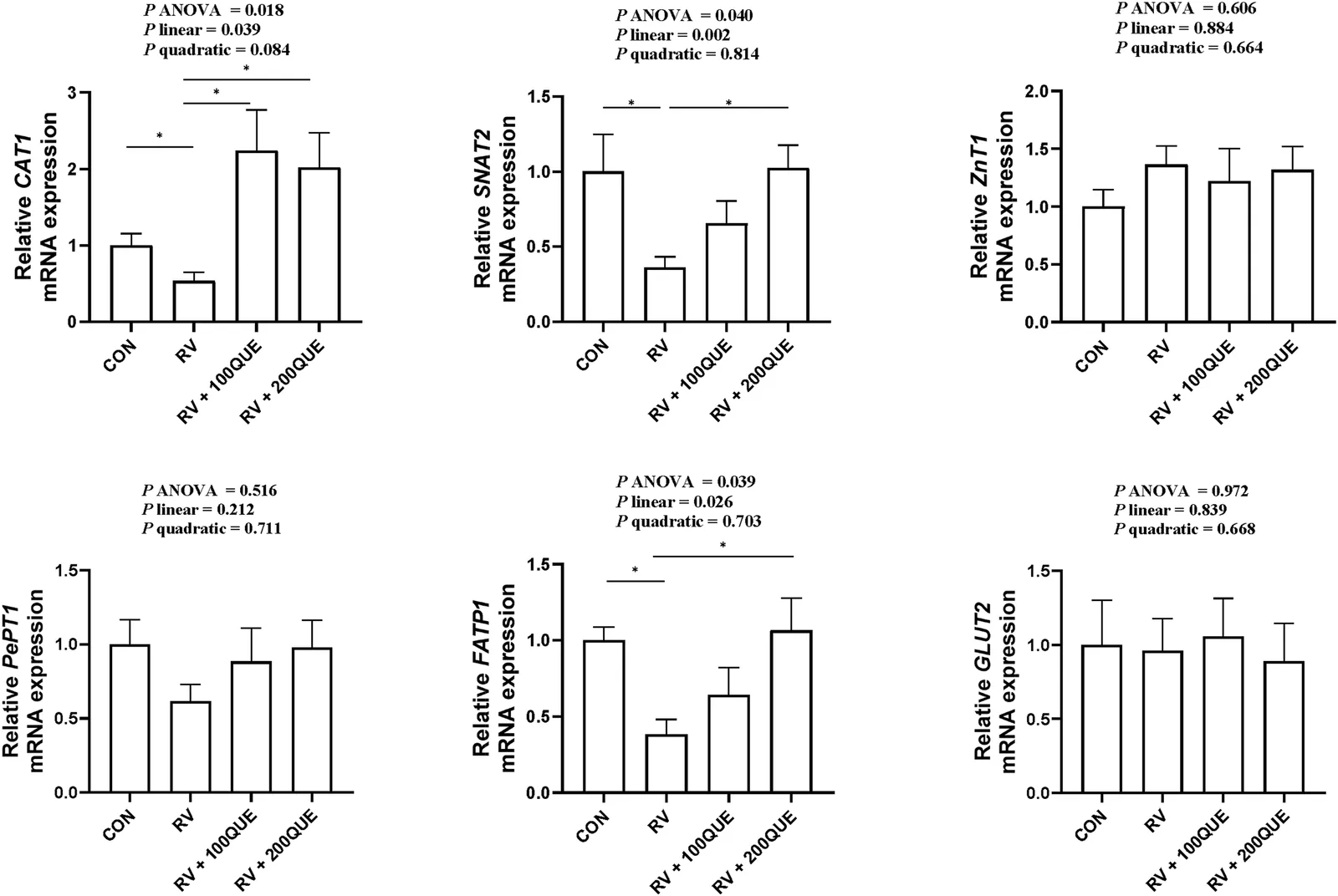
Dietary quercetin (QUE) modulates jejunal nutrient transporter-related gene expression in weaned pigs challenged with rotavirus (RV). CON, non-challenged pigs fed the basal diet; RV, RV-challenged pigs fed the basal diet; RV  +  100QUE, RV-challenged pigs fed the basal diet supplemented with 100 mg/kg QUE; RV +  200QUE, RV-challenged pigs fed the basal diet supplemented with 200 mg/kg QUE. The ANOVA *P*-value represents the overall comparison among all four treatment groups. Linear and quadratic *P*-values were obtained from polynomial contrasts among RV-challenged pigs receiving 0, 100, or 200 mg/kg QUE. Data are presented as mean ± standard error of the mean (SEM); *n* = 8. ∗*P* < 0.05.

### 3.7 Effects of dietary quercetin supplementation on intestinal barrier integrity of weaned pigs challenged with RV

Serum DAO activity and D-lactate concentration are presented in Table 7. Compared with the CON group, RV challenge increased DAO activity and D-lactate content (*P* < 0.05). Within RV-challenged piglets, dietary supplementation with 200 mg/kg quercetin decreased DAO activity compared with the RV group (*P* = 0.030), and dietary supplementation with 200 mg/kg quercetin decreased D-lactate content (*P* < 0.001). Moreover, under RV challenge, DAO activity and D-lactate content decreased linearly with increasing quercetin dose (linear *P* < 0.05), with D-lactate also exhibiting a significant quadratic response (quadratic *P* = 0.004).

**Table 7:** Effects of dietary quercetin (QUE) supplementation on serum diamine oxidase (DAO) activity and D-lactate concentration in weaned pigs challenged with rotavirus (RV).

Item	Treatments^1^				SEM	*P-*value^2^		
	CON	RV	RV + 100 QUE	RV + 200 QUE		ANOVA	Linear	Quadratic
DAO, U/mL	6.33^b^	13.72^a^	10.08^ab^	8.75^b^	0.819	0.030	0.034	0.511
D-lactate, μmol/L	12.45^2^	19.14^1^	18.86^1^	14.12^2^	0.618	<0.001	<0.001	0.004

SEM = standard error of the mean.

Values in the same row with different superscript letters differ significantly (*P* ＜ 0.05); *n* = 8.

1 CON, non-challenged pigs fed the basal diet; RV, RV-challenged pigs fed the basal diet; RV  +  100QUE, RV-challenged pigs fed the basal diet supplemented with 100 mg/kg QUE; RV  +  200QUE, RV-challenged pigs fed the basal diet supplemented with 200 mg/kg QUE.

2 The ANOVA *P*-value represents the overall comparison among all four treatment groups. Linear and quadratic *P*-values were obtained from polynomial contrasts among RV-challenged pigs receiving 0, 100, or 200 mg/kg QUE.

### 3.8 Effects of dietary quercetin supplementation on jejunal epithelial tight junction integrity of weaned pigs challenged with RV

Fig. 3 illustrates phenotypic and transcriptional changes related to jejunal tight junction integrity, including ZO-1 immunofluorescence staining and tight junction related mRNA expression. Compared with the CON group, RV challenge weakened ZO-1 immunoreactivity along the jejunal epithelial lining. Consistently at the transcriptional level, RV challenge decreased relative *ZO-*1 mRNA expression (*P* = 0.037) and showed a decreasing trend in relative *Occludin* mRNA expression (*P* = 0.087), suggesting compromised tight junction integrity. Under RV challenge, dietary supplementation with 100 and 200 mg/kg quercetin increased jejunal ZO-1 fluorescence signals compared with the RV group (*P* < 0.001). In parallel, dietary supplementation with 200 mg/kg quercetin increased relative *ZO-1* and *Occludin* mRNA expression (*P* < 0.05), while dietary supplementation with 100 and 200 mg/kg quercetin increased relative *Claudin1* mRNA expression (*P* = 0.004).

**Fig. 3:**
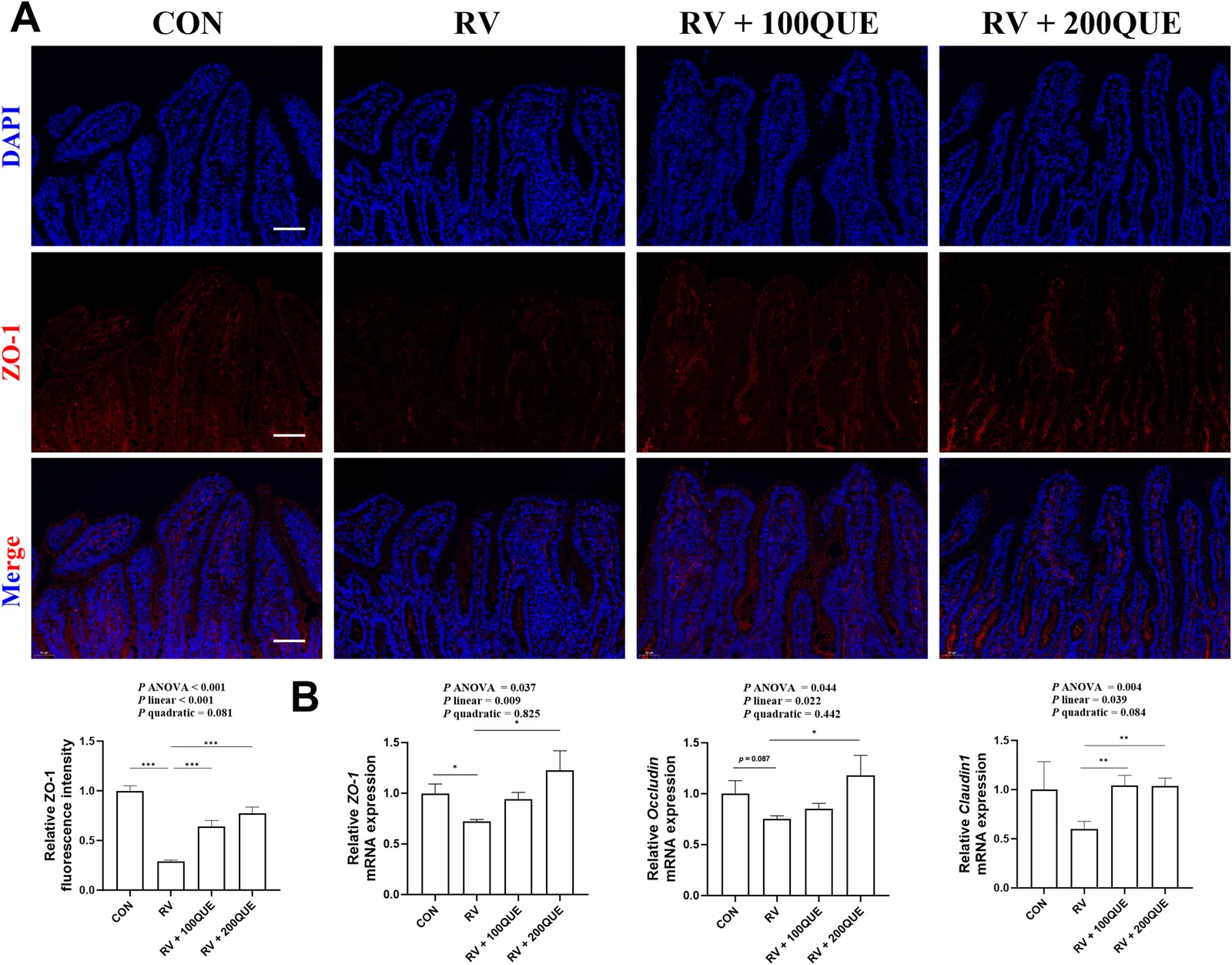
Dietary quercetin (QUE) improves jejunal tight-junction integrity in weaned pigs challenged with rotavirus (RV). (A) Representative immunofluorescence images of zonula occludens-1 (ZO-1; red) and nuclei stained with 4′,6-diamidino-2-phenylindole (DAPI; blue) in jejunal sections. Scale bar = 50 μm. (B) Relative mRNA expression of the tight-junction-related genes *ZO-1*, *Occludin*, and *Claudin1* in the jejunum. CON, non-challenged pigs fed the basal diet; RV, RV-challenged pigs fed the basal diet; RV  +  100QUE, RV-challenged pigs fed the basal diet supplemented with 100 mg/kg QUE; RV  +  200QUE, RV-challenged pigs fed the basal diet supplemented with 200 mg/kg QUE. The ANOVA *P*-value represents the overall comparison among all four treatment groups. Linear and quadratic *P*-values were obtained from polynomial contrasts among RV-challenged pigs receiving 0, 100, or 200 mg/kg QUE. Data are presented as mean ± standard error of the mean (SEM); *n* = 8. ∗*P* < 0.05, ∗∗*P* < 0.01, ∗∗∗*P* < 0.001.

### 3.9 Effects of dietary quercetin supplementation on jejunal mucosal sIgA distribution of weaned pigs challenged with RV

Jejunal sIgA immunohistochemical staining and its quantitative indices (mean optical density and positive signal abundance) are shown in Fig. 4. Compared with the CON group, RV reduced jejunal sIgA immunoreactivity, whereas 100 and 200 mg/kg quercetin increased sIgA staining under RV challenge (*P* < 0.001).

**Fig. 4:**
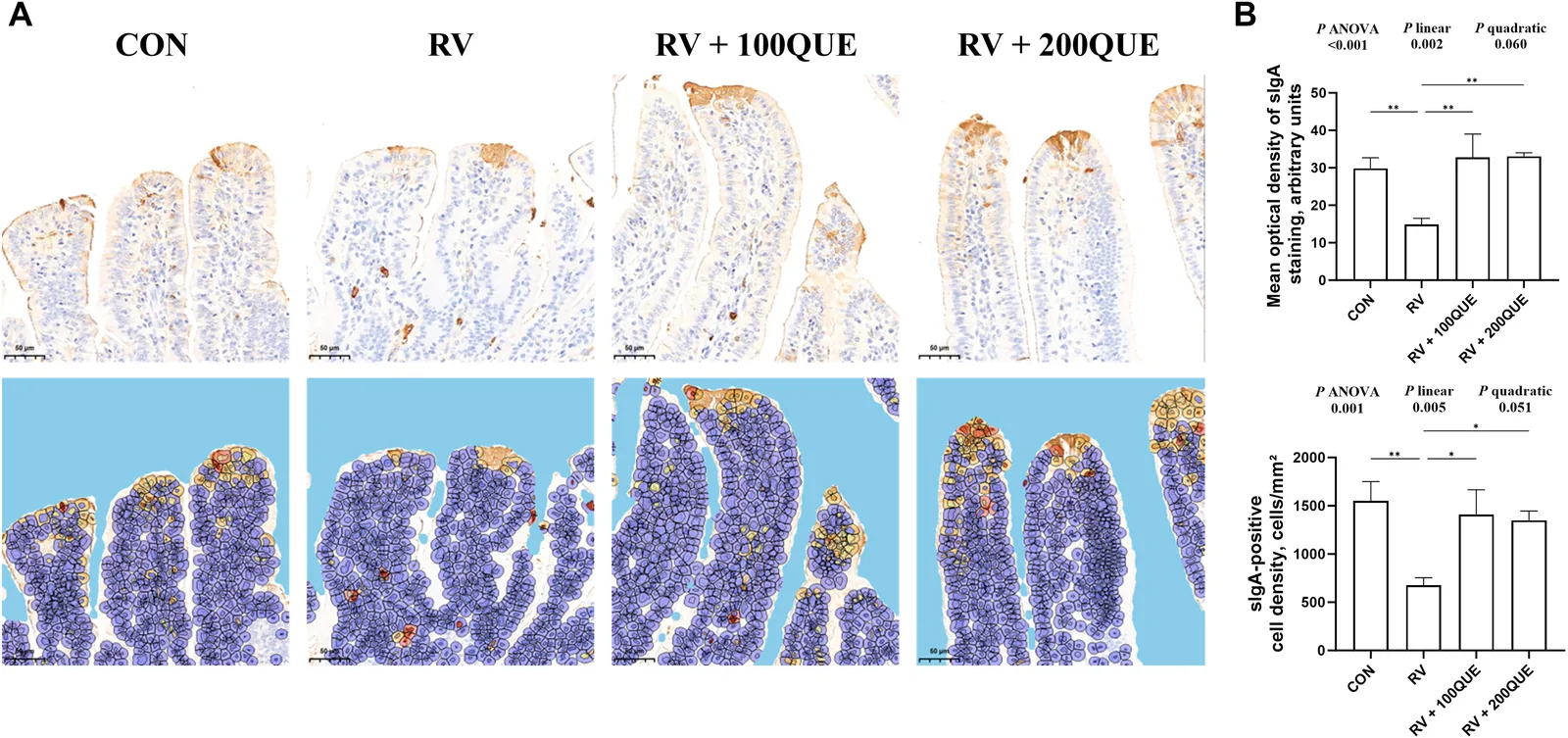
Dietary quercetin (QUE) supplementation restores jejunal mucosal secretory immunoglobulin A (sIgA) responses in weaned pigs challenged with rotavirus (RV). (A) Representative immunohistochemical staining of sIgA (brown) in jejunal sections and the corresponding image-analysis segmentation used for quantification. Scale bar = 50 μm. (B) Quantification of the mean optical density of sIgA staining and sIgA-positive cell density. CON, non-challenged pigs fed the basal diet; RV, RV-challenged pigs fed the basal diet; RV  +  100QUE, RV-challenged pigs fed the basal diet supplemented with 100 mg/kg QUE; RV  +  200QUE, RV-challenged pigs fed the basal diet supplemented with 200 mg/kg QUE. The ANOVA *P*-value represents the overall comparison among all four treatment groups. Linear and quadratic *P*-values were obtained from polynomial contrasts among RV-challenged pigs receiving 0, 100, or 200 mg/kg QUE. Data are presented as mean ± standard error of the mean (SEM); *n* = 8. ∗*P* < 0.05, ∗∗*P* < 0.01.

### 3.10 Effects of dietary quercetin supplementation on jejunal epithelial cell apoptosis of weaned pigs challenged with RV

Fig. 5 illustrates phenotypic and transcriptional changes related to jejunal epithelial apoptosis. Compared with the CON group, RV challenge increased the percentages of early-stage apoptotic cells and total apoptotic cells in jejunal epithelium cells (*P* < 0.05). Consistently at the transcriptional level, RV challenge increased relative *Caspase8*, *BAX*, and *BIM* mRNA expression (*P* < 0.05), while relative *Caspase3* mRNA showed an increasing trend (*P* = 0.070). Under RV challenge, dietary supplementation with 200 mg/kg quercetin reduced the percentages of early-stage apoptotic cells, late-stage apoptotic cells, and total apoptotic cells compared with the RV group (*P* < 0.05). In parallel, dietary supplementation with 100 mg/kg quercetin decreased relative *Caspase8* and *BAX* mRNA expression (*P* < 0.05), and dietary supplementation with 100 and 200 mg/kg quercetin decreased relative *BIM* mRNA expression (*P* = 0.009) relative to the RV group.

**Fig. 5:**
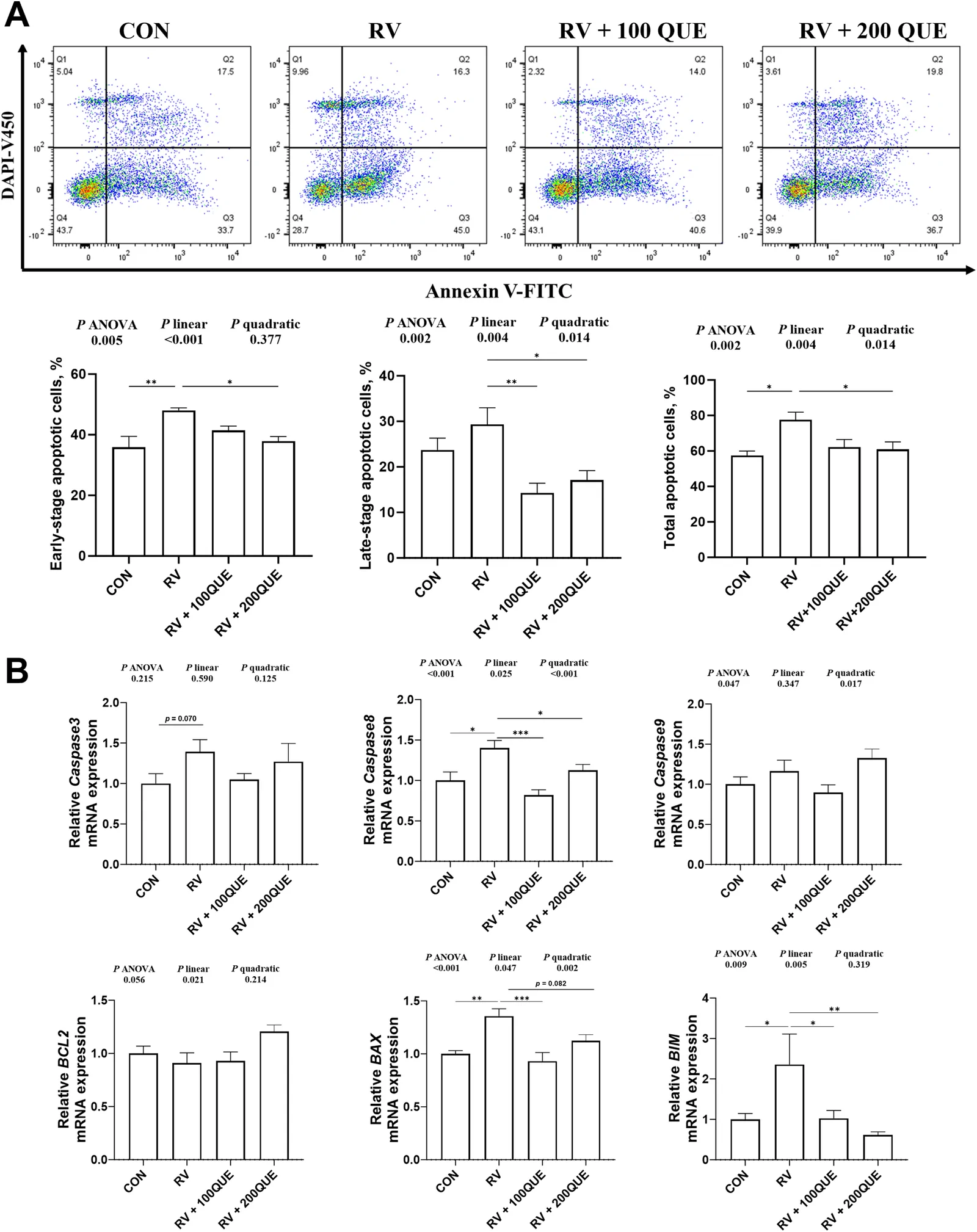
Dietary quercetin (QUE) attenuates jejunal epithelial apoptosis in weaned pigs challenged with rotavirus (RV). (A) Representative flow-cytometric dot plots of Annexin V conjugated to fluorescein isothiocyanate (Annexin V-FITC) and 4′,6-diamidino-2-phenylindole (DAPI) staining. Quantification of early apoptotic cells (Annexin V^+^/DAPI^−^), late apoptotic cells (Annexin V^+^/DAPI^+^), and total apoptotic cells (early + late) is presented. (B) Jejunal mRNA expression of apoptosis-related genes was determined by real-timequantitative PCR (RT-qRCR). CON, non-challenged pigs fed the basal diet; RV, RV-challenged pigs fed the basal diet; RV  +  100QUE, RV-challenged pigs fed the basal diet supplemented with 100 mg/kg QUE; RV  +  200QUE, RV-challenged pigs fed the basal diet supplemented with 200 mg/kg QUE. The ANOVA *P*-value represents the overall comparison among all four treatment groups. Linear and quadratic *P*-values were obtained from polynomial contrasts among RV-challenged pigs receiving 0, 100, or 200 mg/kg QUE. Data are presented as mean ± standard error of the mean (SEM); *n* = 8. ∗*P* < 0.05, ∗∗*P* < 0.01, ∗∗∗*P* < 0.001.

### 3.11 Effects of quercetin on epithelial cell viability, apoptosis, and barrier-related properties in vitro

Building on the in vivo findings that dietary quercetin alleviated RV-induced intestinal injury, in vitro validation was subsequently performed in intestinal epithelial cells to assess the direct effects of quercetin on epithelial viability, apoptosis, and barrier-related properties under RV challenge. Quercetin alone did not reduce cell viability across the tested concentrations (Fig. 6A), whereas RV infection decreased viability; under infection, quercetin improved cell viability (*P* < 0.001; Fig. 6B). Flow cytometry showed that RV increased the percentages of early-stage, late-stage, and total apoptotic cells compared with the control (*P* < 0.05), while quercetin (40 and 80 μmol/L) reduced these apoptotic cell populations under RV challenge (*P* < 0.05; Fig. 6D). Consistently, the mRNA expression heatmap showed that RV upregulated inflammatory cytokine genes (*IL-1β*, *IL-6*, and *TNF-α*) and apoptosis-related genes (*Caspase3*, *Caspase8*, and *Caspase9*) and downregulated tight junction-related genes (*ZO-1*, *Occludin*, and *Claudin1*), whereas quercetin reversed these RV-induced mRNA expression changes (Fig. 6C). In line with these results, RV reduced ZO-1 immunofluorescence signals, which were increased by quercetin relative to the RV group (*P* < 0.001); moreover, RV promoted NF-κB p65 nuclear translocation, while quercetin reduced p65 nuclear localization (*P* < 0.001) (Fig. 6E–G). To extend the translational relevance of the in vitro findings, we performed GSEA using a public transcriptomic dataset of RV-infected human intestinal epithelium, showing significant enrichment of KEGG Apoptosis (KO04210) and NF-κB signaling pathway (KO04064) gene sets (Apoptosis: normalized enrichment score [NES] = 2.0296, false discovery rate [FDR] = 8.78 × 10^−5^; NF-κB: NES = 2.4880, FDR < 0.001) (Fig. 7A–F), which aligns with the apoptosis- and inflammation-related changes observed in porcine intestinal epithelial cells.

**Fig. 6:**
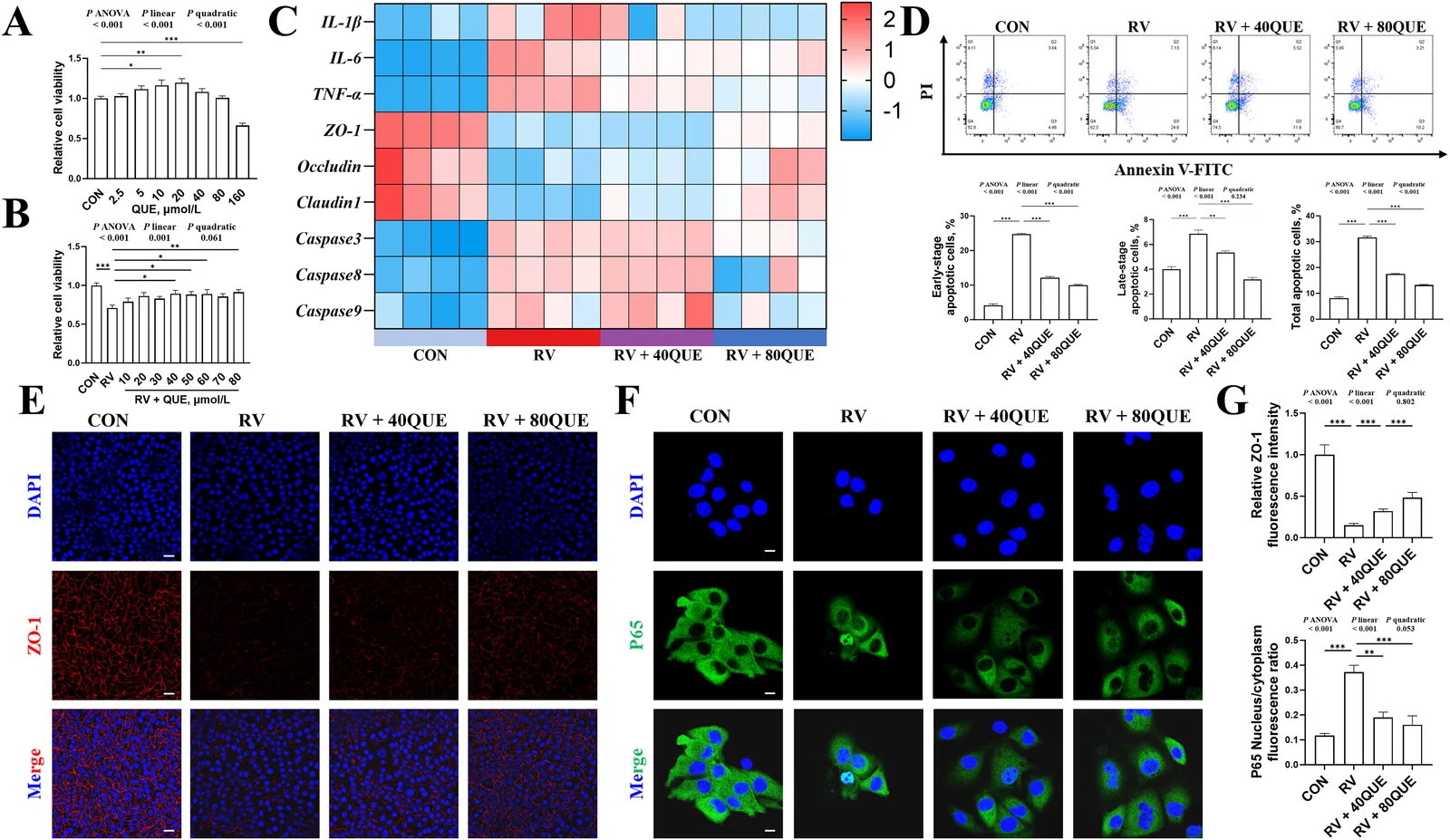
Quercetin (QUE) protects intestinal epithelial cells against rotavirus (RV)-induced inflammatory stress, barrier disruption, and apoptosis, accompanied by reduced nuclear factor-kappa B (NF-κB) activation. (A) Cell viability of intestinal epithelial cells treated with QUE alone at the indicated concentrations. *n* = 6. (B) Cell viability under RV challenge with or without QUE co-treatment. *n* = 6. (C) Heatmap summarizing relative mRNA expression of inflammatory cytokines and barrier/apoptosis-related genes. *n* = 4. (D) Representative flow–cytometric plots of Annexin V conjugated to fluorescein isothiocyanate (Annexin V-FITC) staining and quantification of early-stage, late-stage, and total apoptotic cells. *n* = 4. (E) Immunofluorescence staining of ZO-1 with 4′,6-diamidino-2-phenylindole (DAPI; blue). Scale bar = 25 μm, *n* = 6. (F) Immunofluorescence staining of NF-κB p65 and DAPI, showing RV-induced nuclear translocation and its attenuation by QUE. Scale bar = 10 μm, *n* = 6. (G) Quantification of ZO-1 fluorescence intensity and p65 nuclear localization metrics. CON, untreated control cells; RV, cells infected with RV without QUE treatment; RV  +  40QUE, RV-infected cells treated with 40 μmol/L QUE; RV  +  80QUE, RV-infected cells treated with 80 μmol/L QUE. ANOVA *P* values were obtained from one-way ANOVA across all treatment groups. Linear and quadratic *P*-values were obtained from polynomial trend analyses across QUE concentrations. For RV-challenged experiments, the RV group was considered as 0 μmol/L QUE and the CON group was excluded from the trend analyses. Data are presented as mean ± standard error of the mean (SEM). ∗*P* < 0.05, ∗∗*P* < 0.01, ∗∗∗*P* < 0.001.

**Fig. 7:**
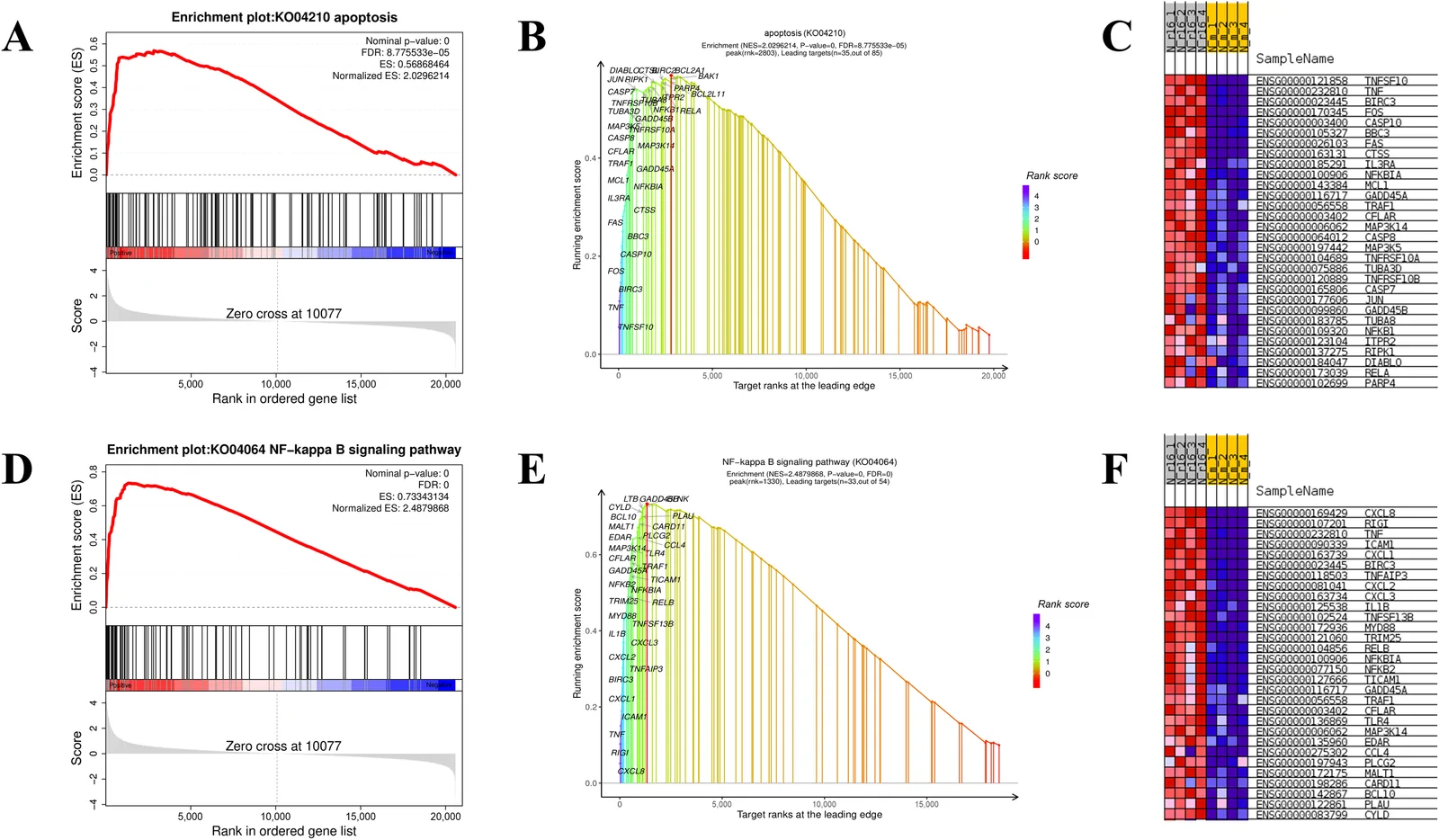
Gene set enrichment analysis of apoptosis and nuclear factor-kappa B (NF-κB) signaling in a public transcriptomic dataset of rotavirus (RV)-infected human intestinal epithelial cells. (A-C) Gene set enrichment analysis (GSEA) of the apoptosis pathway (KEGG KO04210) in a public human intestinal epithelial RV transcriptome, with leading-edge gene visualization. N_m_1–N_m_4 indicate the control group, and N_r16_1–N_r16_4 indicate the RV infected group. (D-F) The GSEA of the NF-κB signaling pathway (KEGG KO04064) with leading-edge gene visualization. FDR = false discovery rate.

## 4 Discussion

Rotavirus is an important enteric pathogen that can cause acute intestinal dysfunction in weaned pigs, thereby reducing growth efficiency and overall production performance (Fan et al., 2021; Vlasova et al., 2017). Quercetin, a widely distributed plant flavonoid, has been proposed as a functional dietary bioactive that may enhance host resilience during infectious stress (Wang et al., 2025). In the present study, quercetin supplementation did not affect ADFI or ADG before RV challenge, which is consistent with previous evidence showing that 200 mg/kg quercetin does not consistently improve growth performance under unchallenged conditions (Mao et al., 2024). However, after RV infection, quercetin attenuated the RV-induced increase in F/G, with the most evident response observed at 200 mg/kg. Considering that RV directly injures the intestinal mucosa and impairs digestion, absorption, and barrier function, the improvement in F/G is likely associated with the ability of quercetin to mitigate infection-associated intestinal dysfunction.

At the serum biochemistry and hematology levels, TP and ALB are key indicators of systemic protein status during infection stress (Chethan et al., 2017), and reduced ALB content may reflect inflammation-related impairment of nutrient absorption, barrier integrity, and protein metabolism (Soeters et al., 2019; Wiedermann, 2021). In this study, RV challenge markedly reduced serum TP and ALB contents, whereas quercetin supplementation (100–200 mg/kg) partially prevented these declines, suggesting improved maintenance of circulating protein status under viral stress. Supporting this interpretation, quercetin has been reported to attenuate ALB reduction in a CCl_4_-induced oxidative injury model (Coballase-Urrutia et al., 2013). Beyond protein indices, RV challenge increased serum ALP content, consistent with an enhanced systemic stress response and/or tissue disturbance during infection (Zhou et al., 2022). Quercetin, particularly at 200 mg/kg, attenuated the RV-induced rise in ALP and showed a dose-responsive pattern, indicating a mitigation of infection-related systemic perturbations. Hematological analysis further revealed that RV challenge reduced RBC, HGB, and HCT levels while increasing PLT counts, suggesting infection-associated disturbance in erythrocyte status and platelet-related inflammatory responses. These changes may reflect impaired oxygen-carrying capacity and activation of coagulation–inflammation pathways during viral infection.

Consistently, previous studies have shown that severe viral infection is often accompanied by decreased TP or ALB contents and erythrocyte-related parameters, together with enhanced systemic inflammation and coagulation activation (Chang et al., 2025). Notably, quercetin supplementation restored RBC, HGB, and HCT levels and reduced PLT counts, indicating that quercetin helped alleviate RV-induced hematological disruption and supported the recovery of physiological homeostasis under infection stress.

Rotavirus infection is commonly associated with excessive production of pro-inflammatory cytokines and suppression of anti-inflammatory responses (Wang et al., 2023; Xiang et al., 2025). Consistent with this pattern, RV challenge in the present study increased TNF-α, IL-1β, and IL-6 contents in both serum and jejunum, while reducing IL-10 content, indicating that RV induced both systemic and intestinal inflammatory responses. These inflammatory changes may partly explain the physiological and biochemical disturbances observed above. Quercetin is an immunomodulatory flavonoid that can suppress pro-inflammatory signaling and promote anti-inflammatory mediators (Li et al., 2025; Wang et al., 2025). Consistent with this, quercetin supplementation in the present study reduced TNF-α and IL-6 contents and increased IL-10 content in both serum and jejunum, suggesting that it alleviated RV-induced inflammatory stress. Moreover, the IPEC-J2 cell results provided epithelial-level evidence for this protective effect: quercetin reduced inflammatory mediator expression and inhibited NF-κB p65 nuclear translocation following RV infection. Given that intestinal epithelial cells are key targets of RV infection and active contributors to mucosal cytokine responses (Haber et al., 2017), these findings suggest that quercetin may mitigate RV-induced intestinal inflammation, at least partly, by suppressing NF-κB-mediated inflammatory activation in epithelial cells. However, because mucosal inflammation in vivo also involves immune cells and epithelial–immune interactions, the specific contribution of immune-cell-mediated regulation requires further investigation.

Intestinal villus height and V:C are key indicators of mucosal architecture, epithelial renewal, and absorptive capacity in weaned piglets (Song et al., 2021). In the present study, RV challenge reduced villus height and V:C in the duodenum, jejunum, and ileum, indicating villus injury and impaired epithelial homeostasis. Quercetin supplementation improved these morphological indices under RV challenge, particularly in the jejunum and ileum, suggesting that quercetin helped preserve intestinal mucosal structure and support epithelial repair during infection stress. This finding is consistent with previous reports showing that dietary quercetin increased jejunal villus height in pigs under transport stress and improved villus morphology in weaned piglets with deoxynivalenol-induced intestinal injury (Liu et al., 2023; Zou et al., 2016). In parallel with the morphological damage, RV challenge reduced intestinal disaccharidase activities in a segment-dependent manner, whereas quercetin partially restored these activities, especially maltase activity in the duodenum and jejunum, indicating improved brush-border digestive function. Because nutrient utilization also depends on enterocyte-mediated transport (McCauley et al., 2020), the quercetin induced recovery of jejunal nutrient transporter genes, including *CAT1*, *SNAT2*, and *FATP1*, further suggests enhanced absorptive capacity during RV infection. Although evidence in virus-challenged pigs remains limited, quercetin has been reported to upregulate intestinal nutrient transport-related genes in vivo (Abdel-Latif et al., 2021), supporting its potential role in maintaining intestinal absorptive machinery under stress. Collectively, these improvements in mucosal morphology, brush-border enzyme activity, and nutrient transporter expression provide a functional basis for the enhanced feed efficiency observed in RV-challenged piglets supplemented with quercetin, likely together with its anti-inflammatory and barrier protective effects.

Efficient nutrient utilization requires not only adequate digestion and transport but also the maintenance of an intact intestinal mucosal barrier (Bildstein et al., 2024). In the present study, RV challenge increased serum DAO and D-lactate levels, reduced jejunal ZO-1 fluorescence intensity, and downregulated tight junction-related genes expression, indicating that RV infection impaired epithelial barrier integrity in vivo. Quercetin supplementation attenuated the increases in DAO and D-lactate levels and restored ZO-1 distribution and tight junction gene expression, suggesting that quercetin helped preserve epithelial barrier homeostasis during RV infection. The IPEC-J2 cell results further supported this barrier-protective effect at the epithelial level. Rotavirus infection disrupted ZO-1 organization, enhanced inflammatory mediator expression, and promoted NF-κB p65 nuclear translocation, whereas quercetin reversed these changes by suppressing NF-κB activation and restoring tight junction organization; this suggests that quercetin may help maintain epithelial barrier integrity and reduce paracellular leakage, thereby limiting the entry of luminal microbial products, including D-lactate, into the bloodstream. This interpretation is consistent with previous studies showing that quercetin strengthens epithelial tight junction assembly and preserves barrier integrity under inflammatory stress (Wang et al., 2024; Xiao et al., 2023). Beyond the structural barrier, the mucosal immune barrier also plays an essential role in limiting luminal antigen exposure and maintaining intestinal homeostasis (Langel et al., 2025). As a key mucosal effector, sIgA can restrict microbial adhesion and antigen translocation, thereby reducing antigenic burden and secondary inflammatory amplification (Conrey et al., 2023). Therefore, the increased jejunal sIgA staining observed in quercetin-supplemented piglets under RV challenge suggests enhanced mucosal immune barrier function, which may act together with preservation of tight junction to support intestinal homeostasis.

Inflammation-induced epithelial apoptosis is an important mechanism underlying intestinal barrier disruption during infection. Pro-inflammatory mediators can activate extrinsic apoptotic signaling, leading to epithelial cell loss, villus atrophy, and impaired tight junction continuity, thereby aggravating intestinal permeability and barrier dysfunction (Liu et al., 2020). In the present study, RV challenge increased early and total apoptosis in jejunal epithelial cells and upregulated pro-apoptotic transcripts, indicating enhanced epithelial cell loss during infection. Quercetin has been reported to exert anti-apoptotic effects in intestinal inflammation and infection models, which are often associated with reduced caspase activation and a shift toward pro-survival BCL-2 family signaling (Jia et al., 2021; Sun et al., 2022). Consistent with these findings, quercetin supplementation, particularly at 200 mg/kg, reduced the percentages of early-stage, late-stage, and total apoptotic jejunal epithelial cells and partially reversed RV-induced changes in apoptosis related gene expression, suggesting that quercetin limited infection associated epithelial loss. This protective effect was further supported by the IPEC-J2 cell model, in which quercetin attenuated RV-induced epithelial apoptosis. Together, these results suggest that inhibition of epithelial apoptosis may represent a key mechanism linking the anti-inflammatory activity of quercetin to its beneficial effects on mucosal morphology and barrier integrity. By reducing inflammatory pressure and preserving epithelial cell viability, quercetin may help maintain villus structure, tight junction continuity, and intestinal barrier function during RV challenge.

Taken together, the in vivo, in vitro, and public transcriptomic evidence supports a coordinated model in which RV infection impairs intestinal homeostasis through interconnected inflammatory, apoptotic, and barrier–disruptive processes. This profile aligns with evidence that enteric viral injury is closely linked to inflammatory activation and apoptosis, which can secondarily compromise barrier architecture (Zhang et al., 2020). The enrichment of NF-κB signaling and apoptosis-related gene sets in public transcriptomic datasets from RV-infected human intestinal epithelium further supports the relevance of these pathways to RV-associated epithelial injury. In this context, quercetin appears to mitigate RV-induced intestinal dysfunction by dampening inflammatory activation, limiting epithelial apoptosis, and preserving tight junction integrity, with NF-κB suppression representing a plausible upstream mechanism linking these protective effects. These epithelial-level actions may help explain the improvements in mucosal morphology, digestive enzyme activity, nutrient transporter expression, and feed efficiency observed in RV-challenged piglets. Nevertheless, cross-species transcriptomic inference and in vitro epithelial models cannot fully recapitulate the complexity of in vivo mucosal immune networks, microbiota-derived metabolism, and dynamic nutrient fluxes. Therefore, while the present findings support a direct epithelial contribution to the protective effects of quercetin, definitive pathway validation in vivo will require targeted mechanistic studies.

## 5 Conclusions

In summary, dietary quercetin supplementation improved feed efficiency and attenuated RV-induced intestinal injury in weaned pigs. These effects were accompanied by a more favorable cytokine profile, reduced epithelial apoptosis, and enhanced intestinal barrier function. The findings support quercetin as a practical nutritional strategy to mitigate RV-induced intestinal injury in weaned pigs.

## Credit Author Statement

**Kang Wang:** Writing – original draft, Formal analysis, Data curation, Conceptualization. **Zhentao He:** Writing – original draft, Formal analysis, Data curation, Conceptualization. **Kunhong Xie:** Supervision, Formal analysis, Data curation, Conceptualization. **Xiang Li:** Supervision, Formal analysis, Data curation, Conceptualization. **Aibing Yu:** Writing – review & editing, Project administration, Methodology. **Yan Li:** Supervision. **Yuheng Luo:** Supervision. **Ping Zheng:** Validation. **Hui Yan:** Validation. **Xiangbing Mao:** Data curation. **Jun He:** Writing – review & editing, Project administration, Methodology, Funding acquisition.

## Declaration of competing interest

We declare that we have no financial and personal relationships with other people or organizations that can inappropriately influence our work, and there is no professional or other personal interest of any nature or kind in any product, service and/or company that could be construed as influencing the content of this paper. The authors declare the following financial interests/personal relationships which may be considered as potential competing interests: Aibing Yu is currently employed by Newhope Co., Ltd., Chengdu, Sichuan, China. Jun He is a Youth Editorial Board Member for *Animal Nutrition* and was not involved in the editorial review or the decision to publish this article.
