# Supplementary material for: Effects of dietary quercetin supplementation on growth performance and intestinal function in weaned pigs challenged by rotavirus
Source: Anim Nutr. 2026 Sep 29;27:578–94. doi: 10.1016/j.aninu.2026.06.005 (PMC13757500; doi:10.1016/j.aninu.2026.06.005)
Supplement: Supplementary file 1 [file mmc1.docx]

**Abbreviations and full names of genes and proteins.**

| **Abbreviations** | **Full names** |
| --- | --- |
| **Genes** |  |
| *BAX* | BCL2-associated X protein |
| *BCL2* | B-cell lymphoma 2 |
| *BIM* | BCL2-interacting mediator of cell death |
| *Caspase3* | Cysteinyl aspartate specific proteinase-3 |
| *Caspase8* | Cysteinyl aspartate specific proteinase-8 |
| *Caspase9* | Cysteinyl aspartate specific proteinase-9 |
| *CAT1* | Cationic amino acid transporter 1 |
| *FATP1* | Fatty acid transport protein 1 |
| *GLUT2* | Glucose transporter 2 |
| *IL-1β* | Interleukin-1 beta |
| *IL-6* | Interleukin-6 |
| *PePT1* | Peptide transporter 1 |
| *SNAT2* | Sodium-coupled neutral amino acid transporter 2 |
| *TNF-α* | Tumor necrosis factor-alpha |
| *VP4* | Viral protein 4 |
| *VP7* | Viral protein 7 |
| *ZnT1* | Zinc transporter 1 |
| *ZO-1* | Zonula occludens-1 |
| **Proteins** |  |
| DAO | Diamine oxidase |
| IL-1β | Interleukin-1 beta |
| IL-6 | Interleukin-6 |
| IL-10 | Interleukin-10 |
| NF-κB p65 | Nuclear factor-kappa B p65 |
| sIgA | Secretory immunoglobulin A |
| TNF-α | Tumor necrosis factor alpha |
| VP6 | Viral protein 6 |
| ZO-1 | Zonula occludens-1 |

**Table S1 Sequence verification of *VP7* and *VP4* in the working rotavirus stock.**

| Genes | Reference length, bp | Sequenced length, bp | Nucleotide identity, % | Nucleotide substitutions | Predicted amino-acid changes | Indel |
| --- | --- | --- | --- | --- | --- | --- |
| *VP7* | 1008 | 1008 | 99.60 | C22A; A232G; G464A; T546C | C22A located in 5′ leader; N69D; G146E; Y173Y | No |
| *VP4* | 2336 | 2336 | 99.57 | G81A; A434C; G532A; A533G; T554C; A800G; G1151A; G1177C; C1938T; C2170A | S27S; K145T; D178S; I185T; Y267C; S384N; D393H; D646D; L724I | No |

**Table S2 Primer sequences.**

| Genes | Primers | Sequences (5’-3’) | Product size, bp | Accession numbers |
| --- | --- | --- | --- | --- |
| *VP7* | Forward | CGACTGGCTATCGGATAGCTCC | 1008 | MT025939.1 |
|  | Reverse | CTAGACTCGGTAATAAAAGGCAGCAG |  |  |
| *VP4* | Forward | ATGGCTTCGCTCATTTATAGAC | 2336 | MT025935.1 |
|  | Reverse | ACTACTTACAGTCTACATTGCA |  |  |
| β-Actin | Forward | GCAAATGCTTCTAGGCGGAC | 148 | XM_021086047.1 |
|  | Reverse | GCGTCCATCACAGCTTCTCA |  |  |
| *IL-1β* | Forward | AGCCAGTCTTCATTGTTCAGGT | 132 | NM_214055.1 |
|  | Reverse | TCATCTCTTTGGGGCCATCAG |  |  |
| *IL-6* | Forward | TTCAGTCCAGTCGCCTTCTCC | 97 | NM_214399.1 |
|  | Reverse | TGGCATCACCTTTGGCATCTTC |  |  |
| *TNF-a* | Forward | CATCGTCTCAAACCTCAGAT | 172 | NM_214022.1 |
|  | Reverse | TGGGAGTAGATGAGGTACAG |  |  |
| *GLUT-2* | Forward | TGGGCTAATTTCAGGCTTGGT | 79 | NM_001097417.1 |
|  | Reverse | AAGAGCACCAATAGCACCCC |  |  |
| *CAT1* | Forward | TGCCCATACTTCCCGTCC | 192 | NM_001012613.1 |
|  | Reverse | GGTCCAGGTTACCGTCAG |  |  |
| *FATP1* | Forward | GGAGTAGAGGGCAAAGCAGG | 208 | NM_001083931.1 |
|  | Reverse | AGGTCTGGCGTGGGTCAAAG |  |  |
| *SNAT2* | Forward | CAAGCTGCTCTGAAAAGCCAT | 122 | XM_047788038.1 |
|  | Reverse | CCAAAGGAAGTAGTACCTGGATGA |  |  |
| *ZnT1* | Forward | ACGCTACCACCATTCAACCT | 112 | NM_001139470.1 |
|  | Reverse | CGTGTCCCACAACATTGCTT |  |  |
| *PePT1* | Forward | CAGACTTCGACCACAACGGA | 99 | NM_214347.1 |
|  | Reverse | TTATCCCGCCAGTACCCAGA |  |  |
| *Caspase3* | Forward | GGATTGAGACGGACAGTGGG | 124 | NM_214131.1 |
|  | Reverse | CCGTCCTTTGAATTTCGCCA |  |  |
| *Caspase8* | Forward | GGATCCCAGGATTTGCCTCC | 135 | NM_001031779.2 |
|  | Reverse | CAGGCTCAGGAACTTGAGGG |  |  |
| *Caspase9* | Forward | ACTCAAGCCAGAGGAGCCAT | 150 | XM_003127618.4 |
|  | Reverse | CTGTCTGGACCACAGACATCA |  |  |
| *BAX* | Forward | ACATGGAGCTGCAGAGGATG | 134 | XM_003127290.5 |
|  | Reverse | GGCAAAGTAGAAAAGCGCGA |  |  |
| *BCL2* | Forward | AGCATGCGGCCTCTATTTGA | 120 | XM_021099593.1 |
|  | Reverse | GGCCCGTGGACTTCACTTAT |  |  |
| *BIM* | Forward | TATGCGCCCGGAGATATGGA | 91 | NM_001160074.1 |
|  | Reverse | AGAAAACAGCATTACCCTCCTTG |  |  |
| *Occludin* | Forward | CTACTCGTCCAACGGGAAAG | 158 | XM_005672523.3 |
|  | Reverse | ACGCCTCCAAGTTACCACTG |  |  |
| *ZO-1* | Forward | CAGCCCCCGTACATGGAGA | 114 | XM_021098896.1 |
|  | Reverse | GCGCAGACGGTGTTCATAGTT |  |  |
| *Claudin1* | Forward | TCTTAGTTGCCACAGCATGG | 106 | NM_001244539.1 |
|  | Reverse | CCAGTGAAGAGAGCCTGACC |  |  |

**Table S3 Complete serum biochemical parameters of weaned pigs challenged with rotavirus (RV) and supplemented with quercetin (QUE).**

| Items | Treatments^1^ | | | | SEM | *P-*value^2^ | | |  |
| --- | --- | --- | --- | --- | --- | --- | --- | --- | --- |
|  | CON | RV | RV + 100  QUE | RV + 200  QUE |  | ANOVA | Linear | Quadratic | |
| CREA, μmol/L | 79.90 | 73.68 | 81.15 | 83.15 | 1.692 | 0.257 | 0.068 | 0.230 | |
| GLU, mmol/L | 6.42 | 6.45 | 6.71 | 6.76 | 0.172 | 0.685 | 0.407 | 0.640 | |
| TC, mmol/L | 1.83 | 1.91 | 1.93 | 2.32 | 0.087 | 0.176 | 0.086 | 0.372 | |
| TG, mmol/L | 0.64 | 0.62 | 0.75 | 0.60 | 0.032 | 0.261 | 0.853 | 0.245 | |
| HDL-C, mmol/L | 0.74 | 0.82 | 0.79 | 0.87 | 0.031 | 0.487 | 0.486 | 0.367 | |
| LDL-C, mmol/L | 0.86 | 0.91 | 0.89 | 1.04 | 0.054 | 0.454 | 0.272 | 0.284 | |

CREA = creatinine; GLU = glucose; TC = total cholesterol; TG = triglycerides; HDL-C = high-density lipoprotein cholesterol; LDL-C = low-density lipoprotein cholesterol; SEM = standard error of the mean.

^1^ CON, non-challenged pigs fed the basal diet; RV, RV-challenged pigs fed the basal diet; RV + 100QUE, RV-challenged pigs fed the basal diet supplemented with 100 mg/kg QUE; RV + 200QUE, RV-challenged pigs fed the basal diet supplemented with 200 mg/kg QUE.

**^2^** The ANOVA P-value represents the overall comparison among all four treatment groups. Linear and quadratic P-values were obtained from polynomial contrasts among RV-challenged pigs receiving 0, 100, or 200 mg/kg QUE.

Values within the same row with no superscript letters indicate no significant difference (*P* ≥ 0.05); *n* = 8.

**Table S4 Complete hematological parameters of weaned pigs challenged with rotavirus (RV) and supplemented with quercetin (**QUE)**.**

| Items | Treatments^1^ | | | | SEM | *P-*value^2^ | | |  |
| --- | --- | --- | --- | --- | --- | --- | --- | --- | --- |
|  | CON | RV | RV + 100  QUE | RV + 200  QUE |  | ANOVA | Linear | Quadratic | |
| WBC, ×10⁹/L | 9.23 | 5.96 | 7.36 | 8.55 | 0.553 | 0.192 | 0.072 | 0.928 | |
| Neu#, ×10⁹/L | 8.21 | 6.63 | 6.75 | 7.96 | 0.525 | 0.642 | 0.359 | 0.665 | |
| Lym#, ×10⁹/L | 12.66 | 10.36 | 10.04 | 11.07 | 0.484 | 0.222 | 0.552 | 0.537 | |
| Mon#, ×10⁹/L | 0.78 | 0.84 | 0.71 | 0.79 | 0.042 | 0.806 | 0.766 | 0.378 | |
| Eos#, ×10⁹/L | 0.35 | 0.28 | 0.23 | 0.26 | 0.016 | 0.074 | 0.681 | 0.334 | |
| Bas#, ×10^7^/L | 3.00 | 1.57 | 2.25 | 1.63 | 0.422 | 0.619 | 0.981 | 0.439 | |
| MCV, fL | 57.75 | 56.88 | 57.51 | 56.48 | 0.503 | 0.811 | 0.775 | 0.532 | |
| MCH, pg | 19.74 | 18.92 | 19.55 | 19.18 | 0.167 | 0.340 | 0.672 | 0.277 | |
| MCHC, g/L | 337.6 | 261.1 | 340.1 | 339.3 | 11.52 | 0.051 | 0.212 | 0.333 | |
| RDW-CV, % | 21.29 | 22.64 | 22.48 | 24.31 | 0.447 | 0.112 | 0.188 | 0.361 | |
| RDW-SD, fL | 46.70 | 49.14 | 49.46 | 50.74 | 0.672 | 0.207 | 0.401 | 0.758 | |
| MPV, fL | 10.69 | 9.69 | 9.94 | 9.64 | 0.201 | 0.224 | 0.904 | 0.577 | |
| PDW, % | 15.55 | 15.30 | 15.94 | 15.64 | 0.136 | 0.452 | 0.442 | 0.185 | |
| PCT, % | 0.36 | 0.43 | 0.35 | 0.35 | 0.035 | 0.814 | 0.700 | 0.677 | |

WBC = white blood cell count; Neu# = absolute neutrophil count; Lym# = absolute lymphocyte count; Mon# = absolute monocyte count; Eos# = absolute eosinophil count; Bas# = absolute basophil count; MCV = mean corpuscular volume; MCH = mean corpuscular hemoglobin; MCHC = mean corpuscular hemoglobin concentration; RDW-CV = red cell distribution width (coefficient of variation); RDW-SD = red cell distribution width (standard deviation); MPV = mean platelet volume; PDW = platelet distribution width; PCT = plateletcrit; SEM = standard error of the mean.

^1^ CON, non-challenged pigs fed the basal diet; RV, RV-challenged pigs fed the basal diet; RV + 100QUE, RV-challenged pigs fed the basal diet supplemented with 100 mg/kg QUE; RV + 200QUE, RV-challenged pigs fed the basal diet supplemented with 200 mg/kg QUE.

**^2^** The ANOVA *P*-value represents the overall comparison among all four treatment groups. Linear and quadratic *P*-values were obtained from polynomial contrasts among RV-challenged pigs receiving 0, 100, or 200 mg/kg QUE.

Values within the same row with no superscript letters indicate no significant difference (*P* ≥ 0.05); *n* = 8.

Fig. S1 Sequence verification of *VP7* and *VP4* genes in the working rotavirus group A. (A) Summary of *VP7* and *VP4* sequence verification by third-generation sequencing. The *VP7* sequence was 1008 bp and showed 99.60% nucleotide identity with the reference sequence, while the *VP4* sequence was 2336 bp and showed 99.57% nucleotide identity. No insertion or deletion was detected in either gene. (B) Linear map of nucleotide substitutions identified in *VP7*. Four nucleotide substitutions were detected: C22A, A232G, G464A, and T546C. C22A was located in the 5′ leader region. Based on translation from the predicted ATG start codon at nucleotide position 28, A232G and G464A resulted in N69D and G146E, respectively, whereas T546C was synonymous (Y173Y). (C) Linear map of nucleotide substitutions identified in *VP4*. Ten nucleotide substitutions were detected: G81A, A434C, G532A, A533G, T554C, A800G, G1151A, G1177C, C1938T, and C2170A. These changes corresponded to two synonymous substitutions and seven predicted amino-acid substitutions: K145T, D178S, I185T, Y267C, S384N, D393H, and L724I. The substitutions at nucleotide positions 532 and 533 occurred within the same codon and together resulted in D178S. Red, nonsynonymous substitutions; green, synonymous substitutions; purple, non-coding substitution.

Fig. S2 Representative high-performance liquid chromatography with ultraviolet detection (HPLC-UV) chromatograms of quercetin in standard solution and experimental diets. **(A) Representative chromatogram of the quercetin standard solution, showing the retention time of the quercetin peak. (B) Representative chromatogram of the basal diet, showing no obvious interfering peak at the retention time corresponding to quercetin. (C) Representative chromatogram of the diet supplemented with 100 mg/kg quercetin. (D) Representative chromatogram of the diet supplemented with 200 mg/kg quercetin.**
